# Mechanisms of glacial‐to‐future atmospheric CO_2_ effects on plant immunity

**DOI:** 10.1111/nph.15018

**Published:** 2018-02-09

**Authors:** Alex Williams, Pierre Pétriacq, Roland E. Schwarzenbacher, David J. Beerling, Jurriaan Ton

**Affiliations:** ^1^ Department of Animal and Plant Sciences University of Sheffield Sheffield S10 2TN UK; ^2^ P^3^ Institute for Translational Soil and Plant Biology Department of Animal and Plant Sciences University of Sheffield Sheffield S10 2TN UK; ^3^ biOMICS Facility Department of Animal and Plant Sciences University of Sheffield Sheffield S10 2TN UK

**Keywords:** Arabidopsis, CO_2_, defence signalling, glycolate oxidase, photorespiration, plant immunity, priming

## Abstract

- The impacts of rising atmospheric CO_2_ concentrations on plant disease have received increasing attention, but with little consensus emerging on the direct mechanisms by which CO_2_ shapes plant immunity. Furthermore, the impact of sub‐ambient CO_2_ concentrations, which plants have experienced repeatedly over the past 800 000 yr, has been largely overlooked.
- A combination of gene expression analysis, phenotypic characterisation of mutants and mass spectrometry‐based metabolic profiling was used to determine development‐independent effects of sub‐ambient CO_2_ (*sa* CO_2_) and elevated CO_2_ (*e*CO_2_) on Arabidopsis immunity.
- Resistance to the necrotrophic *Plectosphaerella cucumerina* (*Pc*) was repressed at *sa* CO_2_ and enhanced at *e*CO_2_. This CO_2_‐dependent resistance was associated with priming of jasmonic acid (JA)‐dependent gene expression and required intact JA biosynthesis and signalling. Resistance to the biotrophic oomycete *Hyaloperonospora arabidopsidis* (*Hpa*) increased at both *e*CO_2_ and *sa* CO_2_. Although *e*CO_2_ primed salicylic acid (SA)‐dependent gene expression, mutations affecting SA signalling only partially suppressed *Hpa* resistance at *e*CO_2_, suggesting additional mechanisms are involved. Induced production of intracellular reactive oxygen species (ROS) at *sa* CO_2_ corresponded to a loss of resistance in glycolate oxidase mutants and increased transcription of the peroxisomal catalase gene *CAT2*, unveiling a mechanism by which photorespiration‐derived ROS determined *Hpa* resistance at *sa*CO_2_.
- By separating indirect developmental impacts from direct immunological effects, we uncover distinct mechanisms by which CO_2_ shapes plant immunity and discuss their evolutionary significance.

## Introduction

Past and future changes in atmospheric CO_2_ directly impact plant metabolism (Temme *et al*., 2015), with feedbacks on resistance to pests and diseases (Strengbom & Reich, 2006; Lake & Wade, 2009; Vaughan *et al*., 2014; Váry *et al*., 2015; Zhang *et al*., 2015; Mhamdi & Noctor, 2016). Although numerous effects of elevated CO_2_ (*e* CO_2_) on disease resistance have been reported, there is little consistency between studies. Some studies report increased disease susceptibility at *e* CO_2_ (Lake & Wade, 2009; Vaughan *et al*., 2014; Váry *et al*., 2015), while others report no, or stimulatory, effects of *e* CO_2_ on disease resistance (Strengbom & Reich, 2006; Riikonen *et al*., 2008; Pugliese *et al*., 2012; Zhang *et al*., 2015; Mhamdi & Noctor, 2016). These discrepancies may arise from differences in *e* CO_2_ concentrations, the duration of *e* CO_2_ exposure, the method of disease quantification, species‐specific adaptations to CO_2_ or a combination of all these factors. Furthermore, biotrophic and necrotrophic pathogens are rarely compared within the same study, providing limited information of how distinct components of the plant immune system respond to *e* CO_2_. To date, various mechanisms by which CO_2_ alters disease resistance have been proposed, ranging from changes in leaf nutrition (Strengbom & Reich, 2006), stomatal density (Lake & Wade, 2009) and pathogen‐specific adaptations to altered host metabolism (Váry *et al*., 2015). Recent evidence suggests a mechanism whereby *e* CO_2_ primes pathogen‐induced production of defence regulatory hormones, such as salicylic acid (SA) and jasmonic acid (JA) (Zhang *et al*., 2015; Mhamdi & Noctor, 2016), which control defences against biotrophic and necrotrophic pathogens, respectively (Thomma *et al*., 1998). Surprisingly, however, most studies do not take into account the stimulatory effects of CO_2_ on plant development (Temme *et al*., 2015), despite evidence that developmental stage can have a profound impact on SA‐dependent and ethylene‐dependent defences (Kus *et al*., 2002; Shibata *et al*., 2010).

Knowledge about the effects of sub‐ambient CO_2_ (*sa*CO_2_) on plant immunity is limited and may give valuable insights into the evolution of plant defence metabolism at typically low CO_2_ (below 200 ppm) during glacial periods over the past 800 000 yr (Temme *et al*., 2015; Galbraith & Eggleston, 2017). While stomatal processes have been implicated in defence at *sa*CO_2_ (Zhou *et al*., 2017), the contribution of *sa*CO_2_ towards post‐invasive plant defence remains unknown. At *sa*CO_2_, net photosynthetic rate decreases as a consequence of photorespiration, along with increased stomatal conductance, increased foliar nitrogen and lower water use efficiency (Temme *et al*., 2013; Li *et al*., 2014). Although it remains unclear whether these changes influence disease resistance, a recent transcriptome study at *sa*CO_2_ revealed enhanced activity of peroxisomal processes that correlate with changes in expression of defence‐related genes (Li *et al*., 2014). For instance, peroxisomal metabolism is stimulated at *sa*CO_2_ (Li *et al*., 2014), which can boost defence through changes in cellular redox homeostasis (Sørhagen *et al*., 2013). The photorespiratory machinery is a major source of intracellular hydrogen peroxide (H_2_O_2_), which plays an important signalling role in plant defence (Chaouch *et al*., 2010). This is further highlighted by the CATALASE‐deficient *cat2* mutant, which is impaired in scavenging of peroxisomal H_2_O_2_ and expresses a constitutive defence phenotype (Chaouch *et al*., 2010). Therefore, it is plausible that *sa*CO_2_ influences plant resistance, but the extent, specificity and regulatory mechanisms remain unknown.

In this study, we have examined the direct impacts of *sa*CO_2_ (200 ppm), *a*CO_2_ (400 ppm) and *e* CO_2_ (1200 ppm) on plant immunity by eliminating confounding effects of CO_2_ on plant development. Using a plant development correction, we show that CO_2_ has differential impacts on resistance against the biotrophic oomycete *Hyaloperonospora arabidopsidis* (*Hpa*) and the necrotrophic fungus *Plectosphaerella cucumerina* (*Pc*). Subsequent molecular and biochemical characterization of CO_2_‐dependent resistance phenotypes uncovered differing mechanisms by which CO_2_ shapes the plant immune system. Apart from priming effects of *e* CO_2_ on hormone‐dependent defences, we provide evidence for a critical role of photorespiration in plant defence at *sa*CO_2_ and discuss possible evolutionary implications.

## Materials and Methods

### Reagents and chemicals

All chemicals and reagents were purchased from Sigma‐Aldrich unless stated otherwise.

### Plant cultivation and growth conditions

*Arabidopsis thaliana* (L.) Heynh. accession Col‐0 was used as wild‐type plant genotype throughout this study, along with Col‐0 mutant lines *npr1‐1* (Cao *et al*., 1997), *sid2‐1* (Wildermuth *et al*., 2001), *jar1‐1* (Staswick, 2002), *aos1‐1* (Przybyla *et al*., 2008), *rbohD/F* (Torres *et al*., 2002), *gox1‐2* (SALK_051930; Alonso *et al*., 2003) and *haox1‐2* (SALK_022285; Alonso *et al*., 2003). Plants were cultivated under short‐day conditions (8.5 h 20°C : 15.5 h 18°C, light : dark; 65% relative humidity). Seeds were stratified for 2 d in the dark at 4°C and planted in 60 ml pots, containing a sand : compost mixture (2 : 3). After 7 d of germination, seedlings were thinned to prevent crowding. Plants were cultivated in climate‐ and CO_2_‐controlled growth cabinets (SGC097.PPX.F; Sanyo Gallenkamp PLC, Leicester, UK) under ambient conditions (*a*CO_2_; 400 ppm, i.e. μl l^−1^), sub‐ambient CO_2_ (*sa*CO_2_; 200 ppm) or elevated CO_2_ (*e* CO_2_; 1200 ppm). Growth chambers were supplemented with compressed CO_2_ (BOC, Guildford, UK) or scrubbed with Sofnolime 797 (AP diving, Helston, UK) to maintain constant CO_2_ at indicated concentrations.

### Plant development correction

Using leaf numbers of 3‐ and 4.5‐wk‐old plants as a proxy of development stage at different CO_2_ regimes (Boyes *et al*., 2001), seed germination at *sa*CO_2_ was started 7 d earlier than at *a*CO_2_, whereas seed germination at *e* CO_2_ was delayed by 3 d in comparison to *a*CO_2_. Development correction (DC) resulted in plants with equal numbers of leaves at all three CO_2_ concentrations at the day of pathogen inoculation (eight‐leaf stage for *Hpa* and 18‐leaf stage for *Pc*; Supporting Information Fig. S1). This experiment was repeated once with comparable results.

### Pathogenicity assays

Due to its sensitivity to age‐related resistance (ARR), assays with *Hpa* (strain WACO9) were conducted with relatively young plants (3 wk old at *a*CO_2_, or eight‐leaf stage). Plants were inoculated with 5 × 10^4^ conidiospores ml^−1^ and left at high humidity. Shoot tissues were collected at 6 or 7 d post‐inoculation (dpi) for trypan blue staining and microscopy analysis of *Hpa* colonisation, as described previously (Luna *et al*., 2012). Briefly, levels of *Hpa* colonisation were assigned to four distinct classes, as illustrated in Fig. S2: (I) no pathogen development; (II) presence of hyphal colonisation; (III) extensive colonisation and presence of conidiophores; and (IV) extensive colonisation and the presence of conidiophores and > 10 oospores. At least 50 leaves from > 15 plants per treatment were used to determine distributions of inoculated leaves across the four *Hpa* colonization classes. Differences in class distributions between genotype–treatment combinations were analysed for statistical significance, using Fisher's exact tests (R, v.3.1.2). To ensure necrotrophic infection, assays with *Pc* (strain BMM) were based on droplet inoculation (6 μl, 5 × 10^6^ spores ml^−1^) on four to six fully expanded leaves of eight plants at the 18‐leaf stage (4.5 wk old at *a*CO_2_), as described previously (Pétriacq *et al*., 2016a). Disease progression was measured as lesion diameters at 13 dpi. Lesion diameters were averaged per plant and treated as one biological replicate. Differences in average lesion diameter per plant between treatments (*n *=* *8) were analysed for statistical significance by ANOVA (R, v.3.1.2). Pathogenicity assays with the *jar1‐1*,* aos1‐1*,* sid2‐1*,* npr1‐1*,* gox1‐2* and *haox1‐2* mutants were repeated at least once with similar results. The results of both the *Hpa* and the *Pc* assays were verified in independent DC experiments with wild‐type plants (Col‐0), using quantitative PCR (Fig. S3). Shoot material was collected at 6 dpi (*n *=* *4) for quantification of *Hpa* biomass; fully expanded leaves were collected at 8 dpi (*n *=* *4) for quantification of *Pc* biomass. The qPCR quantifications of *Hpa* and *Pc* biomass were performed with pathogen‐specific primers (Table S1), using the PCR conditions described by Anderson & McDowell (2015) and Sanchez‐Vallet *et al*. (2010), respectively.

### Gene expression analysis by reverse‐transcriptase qPCR

RNA extraction, cDNA synthesis and relative quantification of gene expression by reverse‐transcriptase qPCR (RT‐qPCR) were performed as described previously (Pétriacq *et al*., 2016a), using gene‐specific primers (Table S1). Basal and hormone‐induced expression of *PR1* (*AT2G14610*) and *VSP2* (*AT5G24770*) were determined in plants of the eight‐leaf stage after spraying shoots with double‐distilled water, 0.1 mM JA (OlChemim, Olomouc, Czech Republic), or 0.5 mM SA, supplemented with 0.01% Silwet L‐77 until imminent runoff. Each biological replicate in these assays consisted of four leaves from four different plants (*n *=* *3). Expression of *CAT2* (*AT4G35090*), *GOX1* (*AT3G14420*) and *HAOX1* (*AT3G14130*) were measured in plants of the eight‐leaf stage, where each biological replicate consisted of shoot material from one plant (*n *=* *5). Differences in relative transcript levels were analysed for statistical significance, using Welch's *t*‐test (R, v.3.1.2). RT‐qPCR assays to quantify *CAT2*,* GOX1* and *HAOX1* gene expression were repeated once with similar results.

### Mass spectrometry analyses

SA and JA were quantified by ultra‐pressure liquid chromatography coupled to quadrupole time of flight mass spectrometry (UPLC‐Q‐TOF), using MS^E^ technology to confirm compound‐specific fragmentation patterns, as detailed in Methods S1. Each biological replicate in these assays consisted of four pooled leaves from different plant (*n *=* *5). Untargeted metabolic profiling by UPLC‐Q‐TOF MS and statistical data analysis were performed as detailed in Methods S1.

### *In situ* detection of reactive oxygen species

Extracellular reactive oxygen species (ROS) were analysed by 3,3′‐diaminobenzidine (DAB) staining (Daudi & O'Brien, 2012), whereas intracellular ROS were visualised by 2′,7′‐dichlorofluorescein diacetate (DCFH‐DA), as described previously (Pétriacq *et al*., 2016b). Each biological replicate in these assays consisted of one individual leaf collected from different plants (*n *=* *10 for DCFH‐DA, *n *=* *5 for DAB). In both cases, mock‐ or *Hpa*‐treated leaves were sampled at 48 h post‐inoculation (hpi). ROS intensities from DAB or DCFH‐DA images were obtained with an Olympus SZX12 binocular microscope (using an HQ510 1p emission filter for DCFH‐DA fluorescence; excitation/emission: 492–495/517–527 nm) and quantified using Adobe Photoshop (v.CS.5), as described previously (Luna *et al*., 2011; Pétriacq *et al*., 2016b).

## Results

### Plant development biases the assessment of CO_2_‐dependent disease resistance

**Figure 1:**
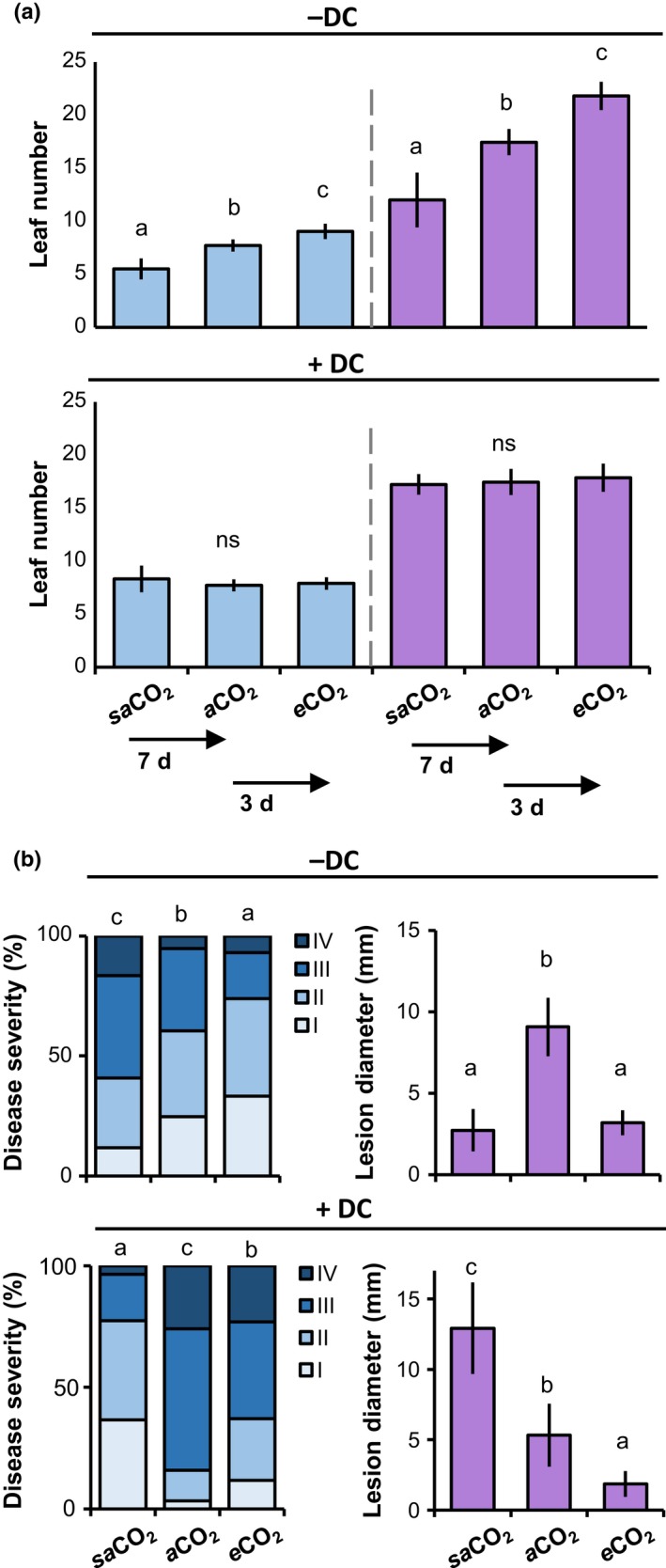
Plant development correction (DC) separates immunological effects of CO_2_ from indirect developmental effects on Arabidopsis resistance. (a) Effect of DC on average leaf numbers in Arabidopsis (Col‐0) at sub‐ambient (*sa* CO_2_; 200 ppm), ambient (*a*CO_2_; 400 ppm) and elevated CO_2_ (*e*CO_2_; 1200 ppm). DC for *sa* CO_2_ was performed by planting seeds 7 d earlier than at *a*CO_2_; DC for *e*CO_2_ was achieved by planting seeds 3 d later than at *a*CO_2_. Upper panel, leaf numbers of 3‐ (left) and 4.5‐ (right) wk‐old plants without DC. Lower panel, leaf numbers after DC. Data represent mean leaf numbers (± SD,* n *=* *10–18) and are representative of two independent experiments. ns, Not significant. (b) Effect of DC on basal resistance against biotrophic *Hyaloperonospora arabidopsidis* (*Hpa*; left) and necrotrophic *Plectosphaerella cucumerina* (*Pc*; right). Shown are relative numbers of leaves (*n *>* *50) in *Hpa* colonization classes of increasing severity (I–IV) at 6 d post‐inoculation (dpi), or average lesion diameters (± SD;* n *=* *8) by *Pc* at 13 dpi. Different letters indicate statistically significant differences (Fisher's exact test; ANOVA with Tukey honest significant difference post‐hoc analysis; *P *<* *0.05). Pathogenicity assays with Col‐0 were repeated several times with comparable outcomes.

To determine the impacts of plant development on CO_2_‐dependent resistance, we first characterised the growth response of Arabidopsis to CO_2_ in different atmospheric CO_2_ concentrations, ranging from 200 ppm (*sa*CO_2_), 400 ppm (*a*CO_2_) to 1200 ppm (*e* CO_2_). Using the number of leaves as a marker for developmental stage (Boyes *et al*., 2001), both 3‐ and 4.5‐wk‐old plants showed enhanced development at *e* CO_2_, and reduced development at *sa*CO_2_, compared to *a*CO_2_ (Fig. 1a, upper panel). To determine whether these developmental effects influence disease resistance, we compared resistance phenotypes against biotrophic *Hpa* and necrotrophic *Pc* with and without correction for plant developmental stage. This DC was achieved by delaying sowing at *e* CO_2_ by 3 d in comparison to plants at *a*CO_2_, while starting plant cultivation at *sa*CO_2_ 7 d earlier compared to plants at *a*CO_2_ (Fig. S1). DC resulted in equal numbers of leaves at all CO_2_ regimes at the time of pathogen inoculation (eight‐leaf stage for *Hpa* and 18‐leaf stage for *Pc*; Fig. 1a, lower panel). Without DC, 3‐wk‐old plants showed increasing levels of *Hpa* resistance at rising CO_2_ concentrations (Fig. 1b, top left), whereas 4.5‐wk‐old plants showed enhanced *Pc* resistance at both *e* CO_2_ and *sa*CO_2_ (Fig. 1b, top right). This pattern of CO_2_‐dependent resistance phenotypes changed upon DC application. While eight‐leaf plants showed enhanced *Hpa* resistance at both *sa*CO_2_ and *e* CO_2_ (Fig. 1b, bottom left), 18‐leaf plants showed increasing levels of *Pc* resistance with rising CO_2_ concentrations (Fig. 1b, bottom right). To confirm the development‐independent effects of CO_2_ on disease resistance, levels of *Hpa* and *Pc* colonization were quantified in an independent DC experiment, using qPCR analysis of pathogen‐specific DNA (Fig. S3). The impact of DC on resistance phenotypes at *sa*CO_2_ and *e* CO_2_ indicates that differences in plant development bias the assessment of CO_2_‐dependent disease resistance against both biotrophic and necrotrophic pathogens. Accordingly, all subsequent experiments were conducted after application of DC.

### Development‐independent effects of eCO_2_ on SA‐ and JA‐dependent resistance

**Figure 2:**
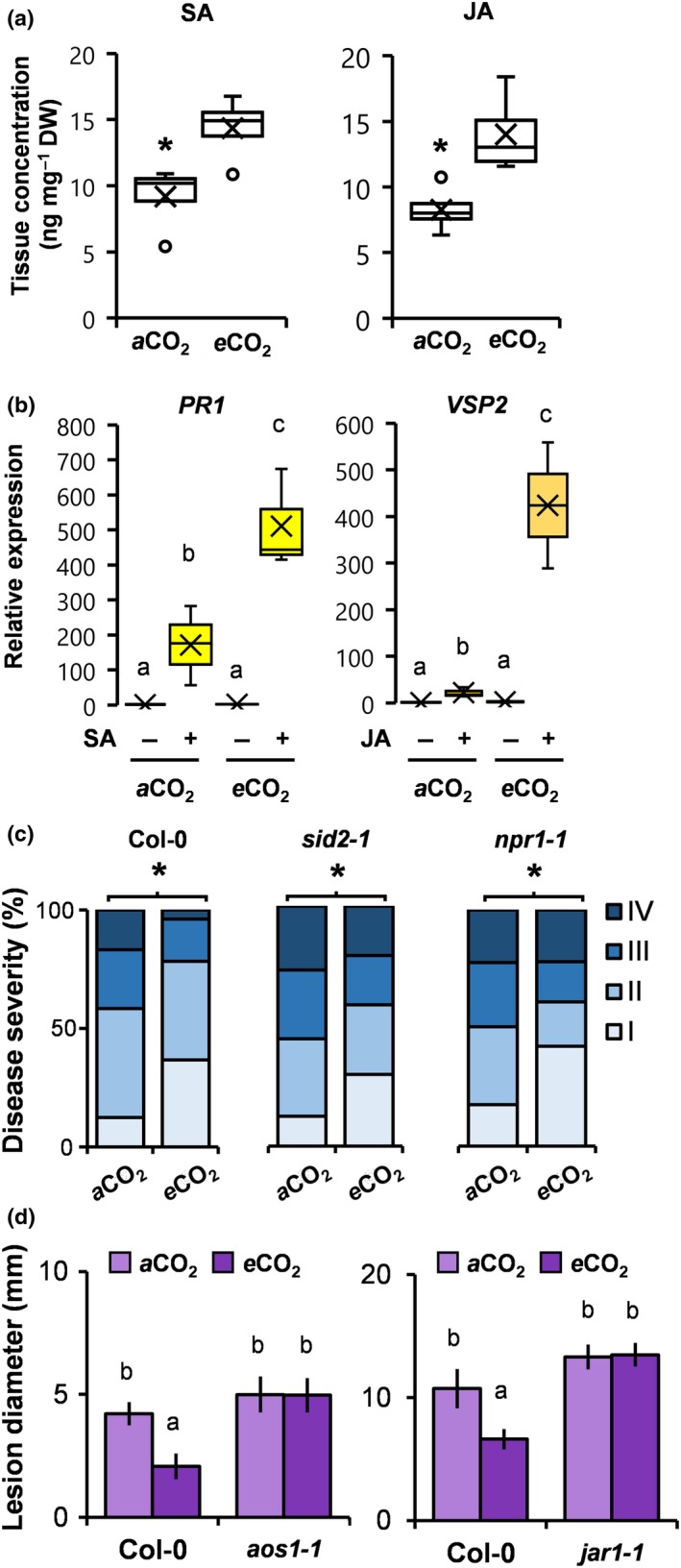
Development‐independent effects of elevated CO_2_ (*e*CO_2_) on salicylic acid (SA)‐ and jasmonic acid (JA)‐dependent defence. (a) Accumulation of SA and JA acids in Arabidopsis (Col‐0) of similar developmental stage (eight‐leaf) at ambient CO_2_ (*a*CO_2_) (400 ppm) and *e*CO_2_ (1200 ppm). Shown are box plots of replicated metabolite quantifications (*n *=* *5; means are indicated by X; outliers outside the 2.5–97.5 percentile interval are indicated by ○). (b) Responsiveness of SA‐ and JA‐inducible genes (*PR1* and *VSP2*, respectively) in eight‐leaf stage plants (Col‐0) at *a*CO_2_ and *e*CO_2_. Shown are box plots of relative transcript levels at 8 and 24 h after treatment (*n *=* *3; means are indicated by X). (c) Effects of *e*CO_2_ on *Hyaloperonospora arabidopsidis* (*Hpa*) resistance in Col‐0, the SA synthesis mutant *sid2‐1* and the SA response mutant *npr1‐1* at the eight‐leaf stage. Shown are relative numbers of leaves (*n *>* *50) in *Hpa* colonisation classes of increasing severity (I–IV) at 6 d post‐inoculation (dpi). (d) Effects of *e*CO_2_ on *Plectosphaerella cucumerina* (*Pc*) resistance in Col‐0, the JA production mutant *aos1‐1* and the *jar1‐1* response mutant at the 18‐leaf stage. Shown are average lesion diameters per plant (± SD;* n *=* *8) of *Pc* at 13 dpi. Asterisks or different letters indicate significant differences between conditions (*P *<* *0.05): (a) Welch's *t*‐test; (c) Fisher's exact test; (b, d) ANOVA with Tukey honest significant difference post‐hoc analysis. Pathogenicity assays with *sid2‐1*,* npr1‐1*,* aos1‐1* and *jar1‐1* were repeated once with similar results.

SA and JA play important roles in plant defence against biotrophic and necrotrophic pathogens, respectively (Thomma *et al*., 1998). To examine the direct (development‐independent) effects of *e* CO_2_ on defence signalling hormones, we used UPLC coupled to tandem MS to quantify SA and JA levels. In comparison to plants at *a*CO_2_, plants at *e* CO_2_ showed a 69.3% and 69.4% increase in accumulation of SA and JA, respectively (Fig. 2a). While increases in hormone levels were not sufficient to induce transcription of the SA‐inducible marker gene *PR1* and the JA‐inducible marker gene *VSP2* directly (Fig. 2b), they were sufficient to prime augmented induction of *PR1* and *VSP2* after exogenous application of 0.5 mM SA and 0.1 mM JA, respectively (Fig. 2b). To determine the contribution of priming of SA‐dependent defence to *e* CO_2_‐induced resistance against *Hpa*, we analysed resistance phenotypes of Arabidopsis mutants impaired in SA production (*sid2‐1*) or response (*npr1‐1*). Although less pronounced than in wild‐type plants (Col‐0), both *sid2‐1* and *npr1‐1* expressed statistically significant levels of *e* CO_2_‐induced resistance against *Hpa* (Fig. 2c). Hence, priming of SA‐dependent defence is not solely responsible for *e* CO_2_‐induced resistance against *Hpa*. To determine the contribution of priming of JA‐dependent defence to *e* CO_2_‐induced resistance against *Pc*, we analysed resistance phenotypes of mutants in JA production (*aos1‐1*) or sensitivity (*jar1‐1*). In contrast to Co1‐0, both *aos1‐1* and *jar1‐1* failed to express elevated *Pc* resistance at *e* CO_2_ (Fig. 2d), indicating that priming of JA‐inducible defence is critically important for *e* CO_2_‐induced resistance against *Pc*.

### Development‐independent resistance at *sa*CO_2_ relies on photorespiration‐derived ROS

**Figure 3:**
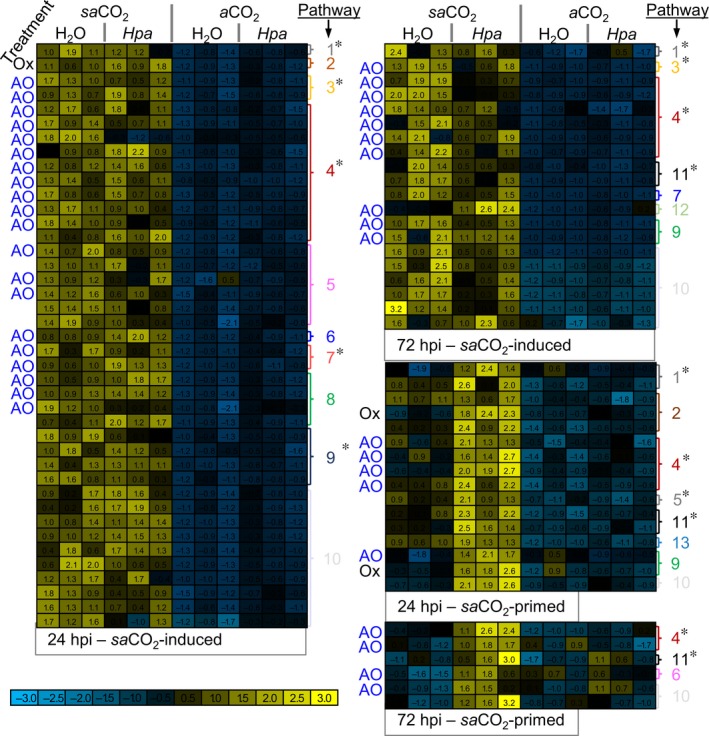
Metabolic profiling of mock‐ and *Hyaloperonospora arabidopsidis* (*Hpa*)‐inoculated Arabidopsis leaves of similar developmental stage at sub‐ambient CO_2_ (*sa* CO_2_) and ambient CO_2_ (*a*CO_2_). Plants of eight‐leaf stage (Col‐0) grown at *sa* CO_2_ (200 ppm) and *a*CO_2_ (400 ppm) were mock‐ or *Hpa*‐inoculated. Methanol extracts from leaves at 24 and 72 h post‐inoculation (hpi) were analysed by UPLC‐Q‐TOF in negative and positive ionisation mode. Normalised ion intensities were filtered for statistically significant differences between treatments, using ANOVA (*P *<* *0.01 + Benjamini–Hochberg false discovery rate correction), followed by two‐way ANOVA (*P *<* *0.01) to select for ion markers that are significantly influenced by CO_2_, *Hpa* or the interaction thereof, at 24 and 72 hpi. Selected markers were subjected to hierarchical clustering (Pearson's correlation). Shown are subclusters of markers showing either enhanced accumulation at *sa* CO_2_ or priming for augmented induction by *Hpa* at *sa* CO_2_. Coloured heat‐maps show normalised ion intensities relative to the average and SD across all samples. Pathways corresponding to putative ion identities are shown on the right of the heat‐maps; antioxidant properties of putative metabolites are indicated by ‘AO’ while putative oxidation products are indicated by ‘Ox’. Pathways with defence properties are marked with an asterisk. Pathway designations are as follows: (1) alkaloids; (2) amino acids; (3) coumarins; (4) flavonoids; (5) lipids; (6) photorespiration; (7) polyphenols; (8) redox; (9) terpenoids; (10) unknown; (11) glucosinolates; (12) polyamines; (13) phytohormones.

Basal resistance against *Hpa* was enhanced at both *e* CO_2_ and *sa*CO_2_ (Fig. 1c). This nonlinear relationship between CO_2_ and *Hpa* resistance suggests involvement of different defence mechanisms at *e* CO_2_ and *sa*CO_2_. Unlike *e* CO_2_ (Fig. 2b), *sa*CO_2_ did not alter basal and SA‐induced *PR1* gene expression (Fig. S4a). Moreover, despite the enhanced disease susceptibility of the SA signalling mutants *sid2‐1* and *nrp1‐1* in comparison to wild‐type plants, both mutants displayed a statistically significant increase in *Hpa* resistance at *sa*CO_2_ in comparison to the same mutant background at *a*CO_2_ (Fig. S4b). Hence, the SA‐dependent defence pathway does not have a critical contribution to *sa*CO_2_‐induced resistance against *Hpa*. To search for alternative mechanisms, we performed untargeted metabolite profiling of mock‐ and *Hpa*‐inoculated plants at 24 and 72 hpi, using UPLC‐Q‐TOF MS (Pétriacq *et al*., 2016b). Unsupervised principal component analysis displayed global metabolic responses, which were affected by both *Hpa* and CO_2_ concentration (Fig. S5). To identify ion markers of *sa*CO_2_‐induced resistance, we applied a stringent pipeline (detailed in Methods S1 and Fig. S6a) to select for ions that are significantly influenced by CO_2_, *Hpa* or the interaction thereof (Fig. S6). Subsequent hierarchical clustering identified ion clusters that are either induced by *sa*CO_2_, or primed by *sa*CO_2_ for augmented induction after subsequent *Hpa* inoculation (Fig. 3). Putative ion marker identification by accurate *m/z* detection revealed enrichment of metabolites involved in cellular redox regulation (NAD metabolism, secondary antioxidant metabolites) and/or defence (glucosinolates, flavonoids, coumarins, alkaloids; Table S2). The cluster containing *sa*CO_2_‐primed markers also included traces of oxidised amino acids (Stadtman & Levine, 2003). Together, these metabolic profiles suggest that plants at *sa*CO_2_ are exposed to enhanced oxidative stress due to increased production of ROS.

**Figure 4:**
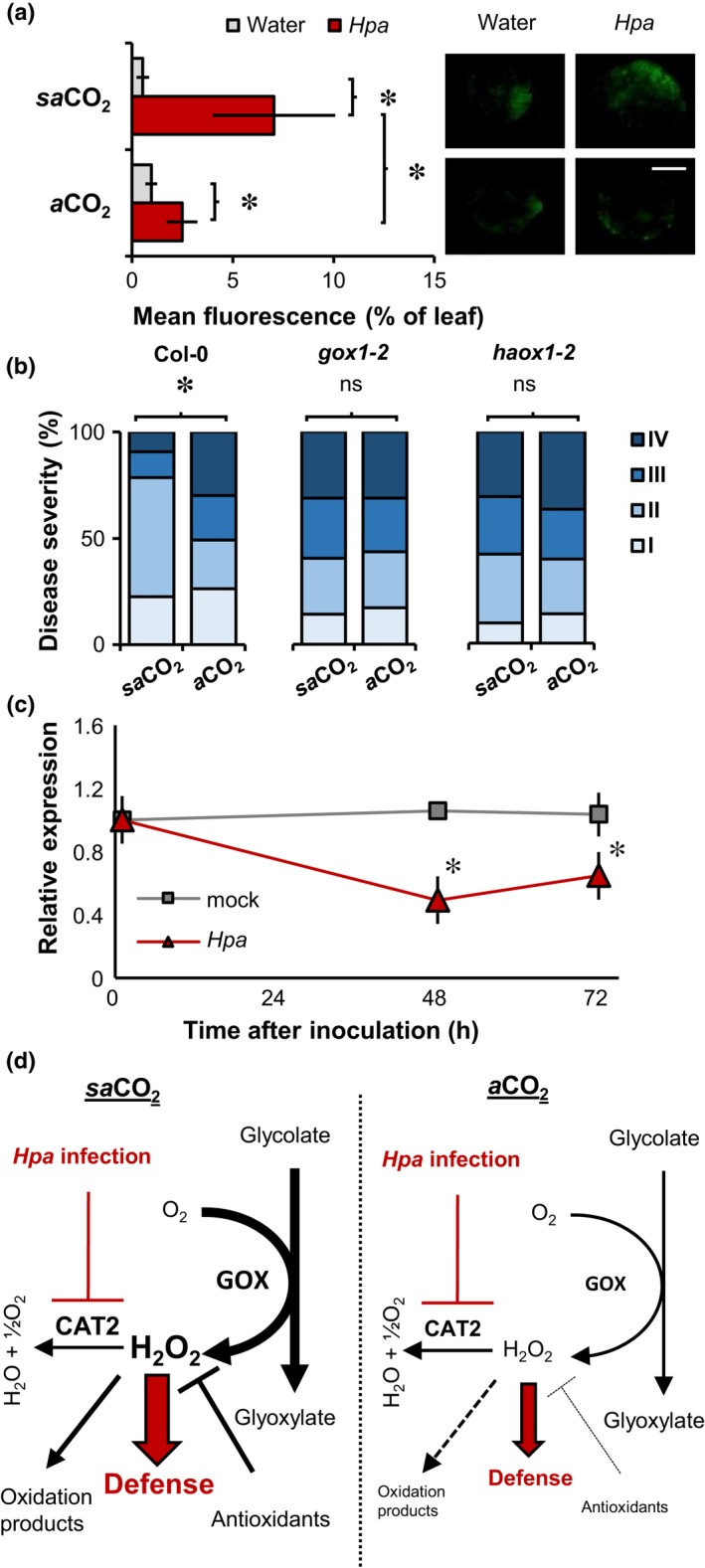
Role of photorespiration in sub‐ambient CO_2_ (*sa* CO_2_)‐induced resistance in Arabidopsis against *Hyaloperonospora arabidopsidis* (*Hpa*). (a) Quantification of intracellular H_2_O_2_ by 2′,7′‐dichlorofluorescein diacetate (DCFH‐DA) staining in plants (Col‐0) of similar developmental stage (eight‐leaf) at *sa* CO_2_ (200 ppm) and *a*CO_2_ (400 ppm). Shown are mean values of the fluorescent proportion of the leaf area (± SD,* n *=* *8–10) at 48 h post‐inoculation (hpi) with water mock or *Hpa*. Insets show representative staining intensities. Bar, 2 mm. (b) Quantification of *Hpa* resistance at *sa* CO_2_ and *a*CO_2_ in wild‐type plants (Col‐0) and glycolate oxidase knock‐down mutants *gox1‐2* and *haox1‐2* at the eight‐leaf stage. Shown are relative numbers of leaves (*n *>* *50) in *Hpa* colonisation classes of increasing severity (I–IV) at 7 d post‐inoculation (dpi). The experiment was repeated with comparable results. (c) Impacts of *Hpa* inoculation on *CAT2* gene expression in 3‐wk‐old Col‐0 at *a*CO_2_ (eight‐leaf stage). Shown are mean values of relative transcript abundance (± SD,* n *=* *5) at different times after water or *Hpa* inoculation. Asterisks indicate statistically significant differences (Welch's *t*‐test; Fisher's exact test; *P *<* *0.05). The experiment was repeated at both *sa* CO_2_ and *a*CO_2_, yielding comparable results (Supporting Information Fig. S9). ns, Not significant. (d) Model explaining the role of photorespiration in priming of reactive oxygen species (ROS)‐dependent defence at *sa* CO_2_. Enhanced photorespiratory activity at *sa* CO_2_ causes increased production of H_2_O_2_ by glycolate oxidase (GOX), which is scavenged by CAT2 and antioxidant metabolites in healthy plants. *Hpa* infection represses transcription of the *CAT2* gene, causing augmented accumulation of GOX‐derived H_2_O_2_ at *sa* CO_2_. Impacts of photorespiration on intracellular H_2_O_2_ are indicated by black arrows. Impacts of *Hpa* on H_2_O_2_‐dependent defence are indicated by red arrows.

As ROS can act as defence signals in plants (Torres *et al*., 2002), we next investigated a possible role for ROS in *sa*CO_2_‐induced resistance. To this end, mock‐ and *Hpa*‐inoculated leaves were stained at 48 hpi with DAB, which predominantly marks extracellular ROS production, because most DAB substrate is immediately oxidised after leaf infiltration by apoplastic H_2_O_2_ and peroxidases (Daudi & O'Brien, 2012). Although *Hpa*‐inoculated leaves showed increased DAB staining intensity, there were no statistically significant differences in extracellular ROS intensities between *sa*CO_2_ and *a*CO_2_ conditions (Fig. S7a,b). Furthermore, the respiratory burst oxidase (RBOH) double mutant *rbohD/F*, which is impaired in stress‐induced production of extracellular ROS (Torres *et al*., 2002), was unaffected in *sa*CO_2_‐induced resistance (Fig. S7c). Hence, extracellular ROS do not play a significant role in *sa*CO_2_‐induced resistance. Subsequently, we stained mock‐ and *Hpa*‐inoculated leaves with DCFH‐DA, which is hydrolysed by intracellular esterases to generate DCF that reacts with intracellular ROS, yielding a fluorescent signal (Sandalio *et al*., 2008). Although *sa*CO_2_ did not increase intracellular ROS accumulation in mock‐inoculated plants, *Hpa*‐inoculated plants at *sa*CO_2_ showed augmented ROS accumulation in comparison with *Hpa*‐inoculated plants at *a*CO_2_ (Fig. 4a). Thus, *sa*CO_2_ primes pathogen‐induced accumulation of intracellular ROS.

A major source of intracellular ROS is photorespiration, which involves production of H_2_O_2_ from oxidation of glycolate by glycolate oxidases (GOXs; Chaouch *et al*., 2010; Rojas *et al*., 2012). Loss‐of‐function mutations in photorespiration cause dramatic growth reduction or lethality at *a*CO_2_ (Timm & Bauwe, 2013), making them unsuitable for evaluation of resistance phenotypes at *a*CO_2_ and *sa*CO_2_. Therefore, we selected single ‘knock‐down’ mutants with T‐DNA insertions in the promotors of *GOX* or *HAOX* (*gox1‐2* and *haox1‐2*, Fig. S8a), which have previously been implicated in Arabidopsis resistance (Rojas *et al*., 2012). Despite the fact that these mutations reduced *GOX1* and *HAOX1* expression by 42.6% and 75.4%, respectively (Fig. S8b), *gox1‐2* and *haox1‐2* showed wild‐type growth phenotypes at *sa*CO_2_ (Fig. S8c). However, unlike wild‐type plants (Col‐0), both mutants failed to express *sa*CO_2_‐induced resistance against *Hpa* (Fig. 4b), indicating a critical role for ROS‐generating GOX function.

In unstressed Arabidopsis plants, GOX‐derived ROS are largely scavenged by the peroxisomal catalase enzyme CAT2 (Chaouch *et al*., 2010). To test whether the augmentation in *Hpa*‐induced ROS production at *sa*CO_2_ (Fig. 4a) is related to changes in *CAT2* expression, we profiled *CAT2* transcript accumulation at different time‐points after mock and *Hpa* inoculation. At both 48 and 72 hpi, *Hpa*‐inoculated plants showed a statistically significant reduction in *CAT2* expression (Fig. 4c), which was apparent at both *a*CO_2_ and *sa*CO_2_ conditions (Fig. S9). Since *sa*CO_2_ boosts photorespiration (Li *et al*., 2014), our results indicate that *Hpa*‐induced *CAT2* repression triggers augmented accumulation of GOX‐derived ROS during infection, which in turn results in enhanced resistance at *sa*CO_2_ (Fig. 4c).

## Discussion

By eliminating bias from indirect developmental effects of CO_2_ on disease resistance, we have identified distinct mechanisms by which CO_2_ shapes plant immunity. There is ample evidence that plant development influences immunity through ARR (Kus *et al*., 2002). ARR in Arabidopsis is effective against (hemi)biotrophic pathogens, including *Pseudomonas syringae* pv *tomato* (*Pst*) and *Hpa* (Kus *et al*., 2002; McDowell *et al*., 2005). When we conducted our experiments without DC, *Hpa* resistance intensified with increasing CO_2_ concentrations (Fig. 1b). DC changed this pattern, revealing that plants of similar developmental stage expressed higher levels of *Hpa* resistance at both *e* CO_2_ and *sa*CO_2_. These results suggest that, in the absence of DC, the resistance‐enhancing effect of *sa*CO_2_ against *Hpa* is masked by low ARR of underdeveloped plants. Interestingly, DC had an opposite effect on CO_2_‐dependent resistance against *Pc*. Without DC, plants showed enhanced resistance at both *sa*CO_2_ and *e* CO_2_, whereas plants of similar developmental stage (i.e. after DC) displayed increasing levels of *Pc* resistance with rising CO_2_ concentrations (Fig. 1b). Thus, without DC, assessment of CO_2_‐dependent resistance against *Pc* is biased by defence mechanisms that are more active at earlier developmental stages. Glucosinolates are known to accumulate to higher concentrations in younger plants (Petersen *et al*., 2002; Brown *et al*., 2003) and are effective against *Pc* (Frerigmann *et al*., 2016). Alternatively, age‐dependent regulation of the JA response could play a role, which is primed in younger plants due to miR156‐dependent repression of JAZ6‐stabilising SPL protein (Mao *et al*., 2017). Taken together, our results show that DC is an effective method to eliminate bias from developmental effects of CO_2_ on disease resistance, enabling a more accurate assessment of mechanisms by which CO_2_ shapes plant immunity.

Previous studies have reported that *e* CO_2_ enhances and/or primes phytohormone‐dependent plant defence (Zhang *et al*., 2015; Mhamdi & Noctor, 2016). However, none of these studies applied DC to eliminate bias from ARR. While some studies transferred plants of similar developmental age from *a*CO_2_ to *e* CO_2_ before pathogen inoculation (Zhang *et al*., 2015), we opted against this method, given it can cause abrupt, and potentially confounding, changes in carbon flux. Furthermore, transferring plants from *a*CO_2_ to *e* CO_2_ before pathogen challenge may neglect the full extent by which *e* CO_2_ affects defence hormone production (Mhamdi & Noctor, 2016). Using DC, we confirmed that *e* CO_2_ enhances basal production of SA and JA (Fig. 2a), causing priming of JA‐ and SA‐dependent gene expression, respectively (Fig. 2b). The JA signalling mutants *aos1‐1* and *jar1‐1* were impaired in expression of *e* CO_2_‐induced resistance against *Pc* (Fig. 2d), indicating a critical contribution of JA‐dependent defence signalling. Conversely, the SA signalling mutants *sid2‐1* and *npr1‐1* were only partially affected in *e* CO_2_‐induced resistance against *Hpa* (Fig. 2c), indicating that priming of SA‐dependent defence is not solely responsible for *Hpa* resistance at *e* CO_2_, which is consistent with previous conclusions regarding *e* CO_2_‐induced resistance against hemi‐biotrophic *Pst* (Zhang *et al*., 2015; Mhamdi & Noctor, 2016). Furthermore, Mhamdi & Noctor (2016) recently reported that *e* CO_2_‐induced resistance to *Pst* is associated with changes in primary metabolism and increased pools of total and oxidised glutathione, while Arabidopsis mutants in glutathione regulation and NADPH‐generating enzymes were affected in *Pst* resistance at *e* CO_2_. Although it is unclear whether these mutants were similarly affected in basal resistance at *a*CO_2_, the study by Mhamdi & Noctor (2016) concluded that oxidative pathways controlling primary metabolism played a role in *e* CO_2_‐induced resistance. Since carbohydrate metabolism and signalling can boost SA‐dependent and SA‐independent defence (Tauzin & Giardina, 2014) by augmenting redox signalling (Morkunas & Ratajczak, 2014), we speculate that *e* CO_2_‐induced resistance in *Hpa* resistance is a consequence of changes in carbohydrate metabolism.

So far, the effects of *sa*CO_2_ on plant disease resistance have received limited attention. Our DC experiments revealed that Arabidopsis expresses enhanced *Hpa* resistance at *sa*CO_2_ (Fig. 1b). Untargeted UPLC‐Q‐TOF analysis revealed that this *sa*CO_2_‐induced resistance was associated with ion clusters displaying constitutively enhanced accumulation and/or primed accumulation after subsequent *Hpa* infection at *sa*CO_2_ (Fig. 3). As these ion clusters were enriched with putative metabolites involved in redox regulation, we explored the importance of ROS in *sa*CO_2_‐induced resistance. While we excluded a role for extracellular ROS (Fig. S7), plants at *sa*CO_2_ showed augmented production of intracellular ROS after *Hpa* inoculation (Fig. 4a). Glycolate oxidation by GOX is a major source of intracellular H_2_O_2_ (Chaouch *et al*., 2010), which probably increases at *sa*CO_2_ due to enhanced photorespiration (Temme *et al*., 2013; Li *et al*., 2014). Moreover, GOX‐derived ROS have been linked to resistance against nonhost pathogens in both Arabidopsis and *Nicotiana benthamiana* (Rojas *et al*., 2012). Indeed, knockdown mutants with reduced transcription of two separate *GOX* genes failed to express enhanced *Hpa* resistance at *sa*CO_2_, indicating a crucial role for photorespiratory ROS. The peroxisomal catalase enzyme, CAT2, scavenges GOX‐derived H_2_O_2_ to mitigate oxidative damage during photorespiration (Chaouch *et al*., 2010). Interestingly, transcriptional profiling of the *CAT2* gene revealed that Arabidopsis reduces *CAT2* expression after *Hpa* inoculation (Figs 4c, S9). Since CAT2 suppresses plant defence (Polidoros *et al*., 2001; Chaouch *et al*., 2010), this pathogen‐induced *CAT2* repression probably reflects an innate immune response to generate defence‐inducing ROS during infection. In this context, we propose that stimulation of photorespiration‐related GOX activity at *sa*CO_2_ primes pathogen‐induced accumulation of intracellular ROS. Subsequent repression of *CAT2* expression following *Hpa* attack results in enhanced accumulation of intracellular ROS, mediating enhanced levels of SA‐independent resistance in comparison to *a*CO_2_‐exposed plants (Fig. 4d).

It is plausible that photorespiration‐derived ROS were key to survival when plants adapted to glacial periods with low atmospheric CO_2_. Reduced growth and plant fecundity at glacial CO_2_ conditions required longer life cycles to maintain reproductive fitness (Ward & Kelly, 2004). Additionally, reduced investment in foliar defence compounds at *sa*CO_2_ would have put plants at a higher risk of pathogen attack (Quirk *et al*., 2013), creating selective pressure for a primed immune system. In addition to limiting the toxicity of 2‐phosphoglycolate, we hypothesise that C_3_ plants benefitted from photorespiration to prime their immune system. This hypothesis may explain why certain C_4_ plants (e.g. maize) have retained photorespiration and GOX activity (Peterhansel & Maurino, 2011). Our study has uncovered a specific link between *sa*CO_2_, GOX‐derived ROS and enhanced immunity. This evidence supports the notion that plants have utilised photorespiratory defence signalling over glacial periods to maintain elevated levels of adaptive broad‐spectrum disease resistance. This may be especially pertinent to Arabidopsis, which evolved under the CO_2_‐limited atmosphere of the Miocene epoch (Beilstein *et al*., 2010). In this context, future initiatives to replace C_3_ metabolism with C_4_ metabolism in major food crops may require careful consideration of the contribution of photorespiration to plant defence.

## Author contributions

A.W., P.P., D.J.B. and J.T. planned and conceived the experiments; A.W., R.E.S., P.P. and J.T. performed the experiments; J.T. and D.J.B. provided reagents, equipment and facilities; A.W., P.P. and J.T. analysed the data; A.W., P.P. and J.T. wrote the paper with feedback from all co‐authors.

## Supporting information

Please note: Wiley Blackwell are not responsible for the content or functionality of any Supporting Information supplied by the authors. Any queries (other than missing material) should be directed to the *New Phytologist* Central Office.

**Fig. S1** Effects of CO_2_ on plant development.
**Fig. S2** Images of *Hpa* colonisation classes.
**Fig. S3** qPCR‐based quantification of *Hpa* and *Pc* biomass.
**Fig. S4** Role of SA signalling in *sa*CO_2_‐induced resistance against *Hpa*.
**Fig. S5** Global metabolic signatures of *Hpa‐*inoculated Arabidopsis at *sa*CO_2_ and *a*CO_2_.
**Fig. S6** Selection of ions that are induced or primed for *Hpa*‐induced accumulation by *sa*CO_2_.
**Fig. S7** Extracellular H_2_O_2_ in *sa*CO_2_‐induced resistance against *Hpa*.
**Fig. S8** Selection of *gox1‐2* and *haox1‐2* mutants.
**Fig. S9** Impacts of *Hpa* inoculation on *CAT2* gene expression at *sa*CO_2_ and *a*CO_2_.
**Table S1** Primers used in this study
**Table S2** Putative identification of metabolic markers detected by UPLC‐Q‐TOF
**Methods S1** Supplemental materials and methods.
