# Supplementary material for: Mechanisms of glacial‐to‐future atmospheric CO 2 effects on plant immunity
Source: New Phytol. 2018 Feb 9;218(2):752–61. doi: 10.1111/nph.15018 (PMC5873421; doi:10.1111/nph.15018)
Supplement: Supplementary file 1 — Fig. S1 Effects of CO2 on plant development. Fig. S2 Images of Hpa colonisation classes. Fig. S3 qPCR‐based quantification of Hpa and Pc biomass. Fig. S4 Role of SA signalling in saCO2‐induced resistance against Hpa. Fig. S5 Global metabolic signatures of Hpa‐inoculated Arabidopsis at saCO2 and aCO2. Fig. S6 Selection of ions that are induced or primed for Hpa‐induced accumulation by saCO2. Fig. S7 Extracellular H2O2 in saCO2‐induced resistance against Hpa. Fig. S8 Selection of gox1‐2 and haox1‐2 mutants. Fig. S9 Impacts of Hpa inoculation on CAT2 gene expression at saCO2 and aCO2. Table S1 Primers used in this study Table S2 Putative identification of metabolic markers detected by UPLC‐Q‐TOF Methods S1 Supplemental materials and methods. [file NPH-218-752-s001.pdf]

New Phytologist Supporting Information Figs S1-S9, Tables S1-S2 and Methods S1

Article title: Mechanisms of glacial-to-future atmospheric CO_2 effects on plant immunity

Authors: Alex Williams, Pierre Pétriacq, Roland E. Schwarzenbacher, David J. Beerling & Jurriaan Ton

Article acceptance date: 26 December 2017

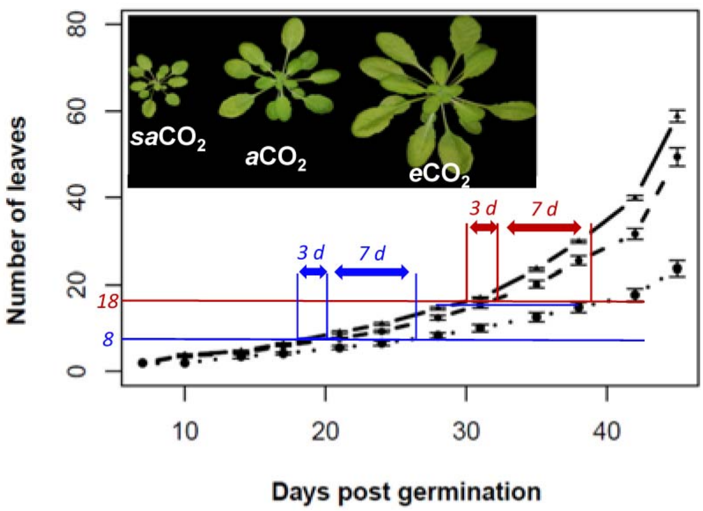

Fig. S1 Effects of CO_2 on plant development. Data represent average leaf numbers (± SE; n = 8) plotted against time (days) at ambient CO_2 (*a*CO_2; 400 ppm; dashed line), subambient CO_2 (*sa*CO_2; 200 ppm; dotted line) and elevated CO_2 (*e*CO_2; 1200 ppm; straight line). Inserts show typical rosette sizes of 4.5-week old plants. Red and blue lines illustrate differences in absolute age at the 8- and 18-leaf stage, respectively. Shown are results from a representative experiment that was repeated twice.

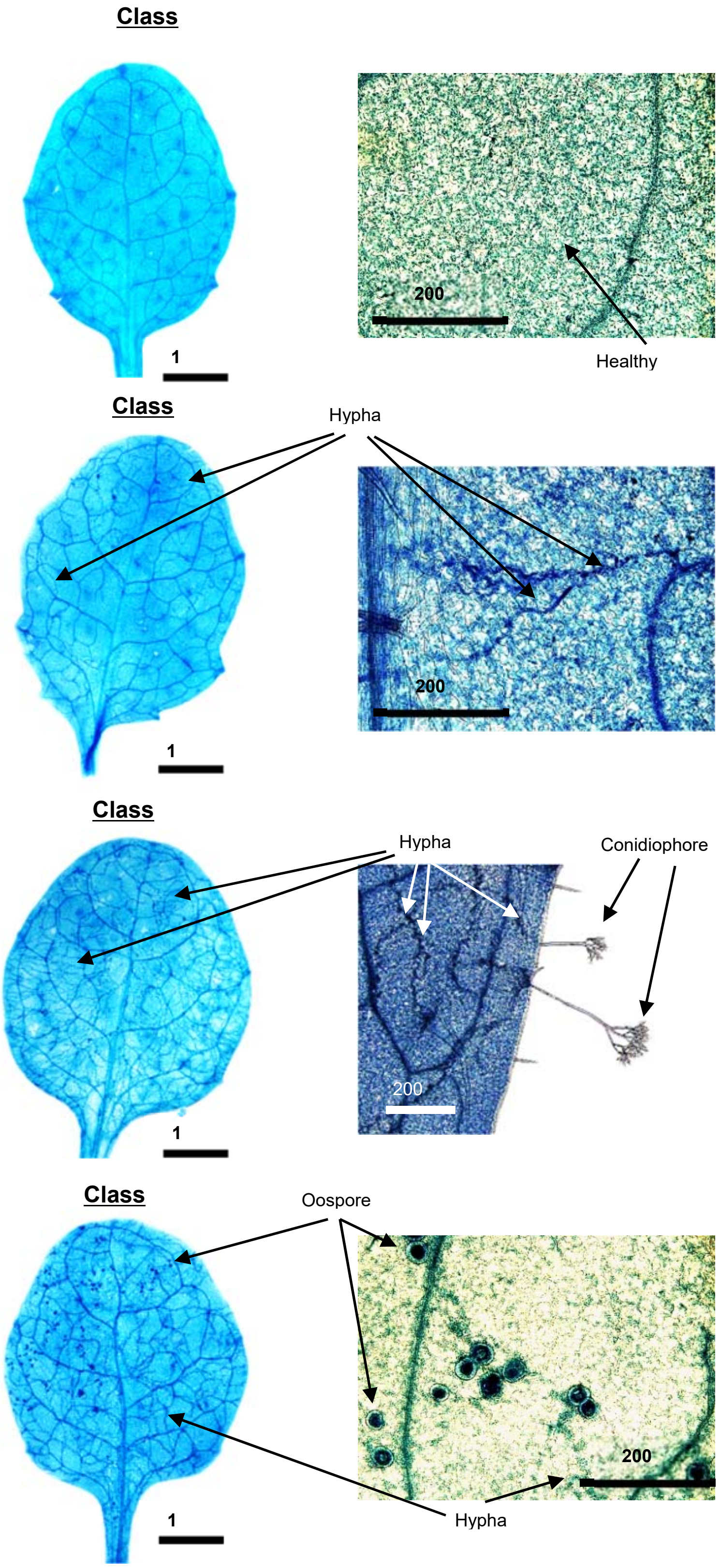

Fig. S2 Representative examples of the four different *Hpa* colonization classes that were used to quantify Arabidopsis resistance. To visualise *Hpa* colonisation, leaves were stained with lactophenol trypan-blue, as described previously (Luna et al. 2012). Class I is defined by a lack of hyphal growth; Class II sustains hyphal development, but not the production of asexual conidiospores; Class III is characterised by extensive hyphal colonisation and the formation of conidiophores and asexual condiospores; Class IV is similar as class III, but with additional formation of sexual oospores (> 10 per leaf). Black bars indicate scales.

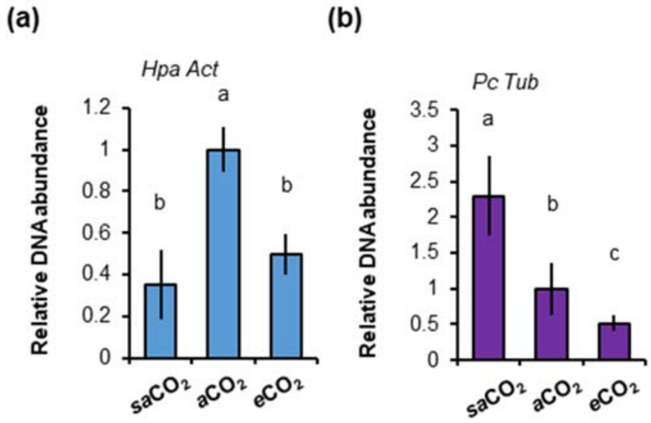

Fig. S3 qPCR-based quantification of pathogen biomass to confirm the development-independent effects of CO_2 on resistance against *Hpa* and *Pc*. (a) Relative quantification of *Hpa* DNA was based on the *Hpa* actin gene (ID: 807716). (b) Relative quantification of *Pc* DNA was based on the *Pc* β-tubulin gene. Data represent relative DNA quantities normalized to Arabidopsis *ACT2* (At3g18780) (± SD, n = 4). Letters indicate statistical differences (ANOVA + Tukey post-hoc analysis; $P < 0.05$). For details about DC and timing of pathogen inoculation, see legend to Fig. 1.

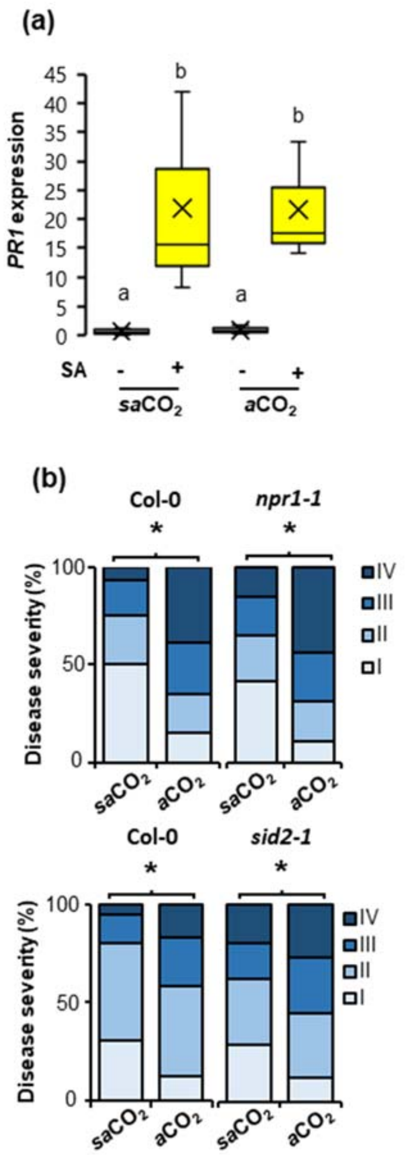

Fig. S4 SA signalling in *sa*CO_2-induced resistance against *Hpa*. (a) Levels of SA-inducible *PR1* gene expression in 8-leaf Col-0 at *sa*CO_2 (200 ppm) and *a*CO_2 (400 ppm). Shown are box plots of relative transcript values (n = 3; means are indicated by X) at 24 hours after treatment. (b) Quantification of *Hpa* resistance at *sa*CO_2 and *a*CO_2 in Col-0, the SA insensitive *npr1-1* mutant, and the SA production mutant *sid2-1* at the 8-leaf stage. Shown are relative numbers of leaves (n > 50) in *Hpa* colonization classes of increasing severity (I–IV) at 7 dpi. Letters - (a) ANOVA with Tukey HSD post hoc analysis - or asterisks - (b) Fisher's exact test - indicate statistically significant differences between conditions ($P < 0.05$). The pathogenicity assays with *sid2-1* and *npr1-1* were repeated with similar results.

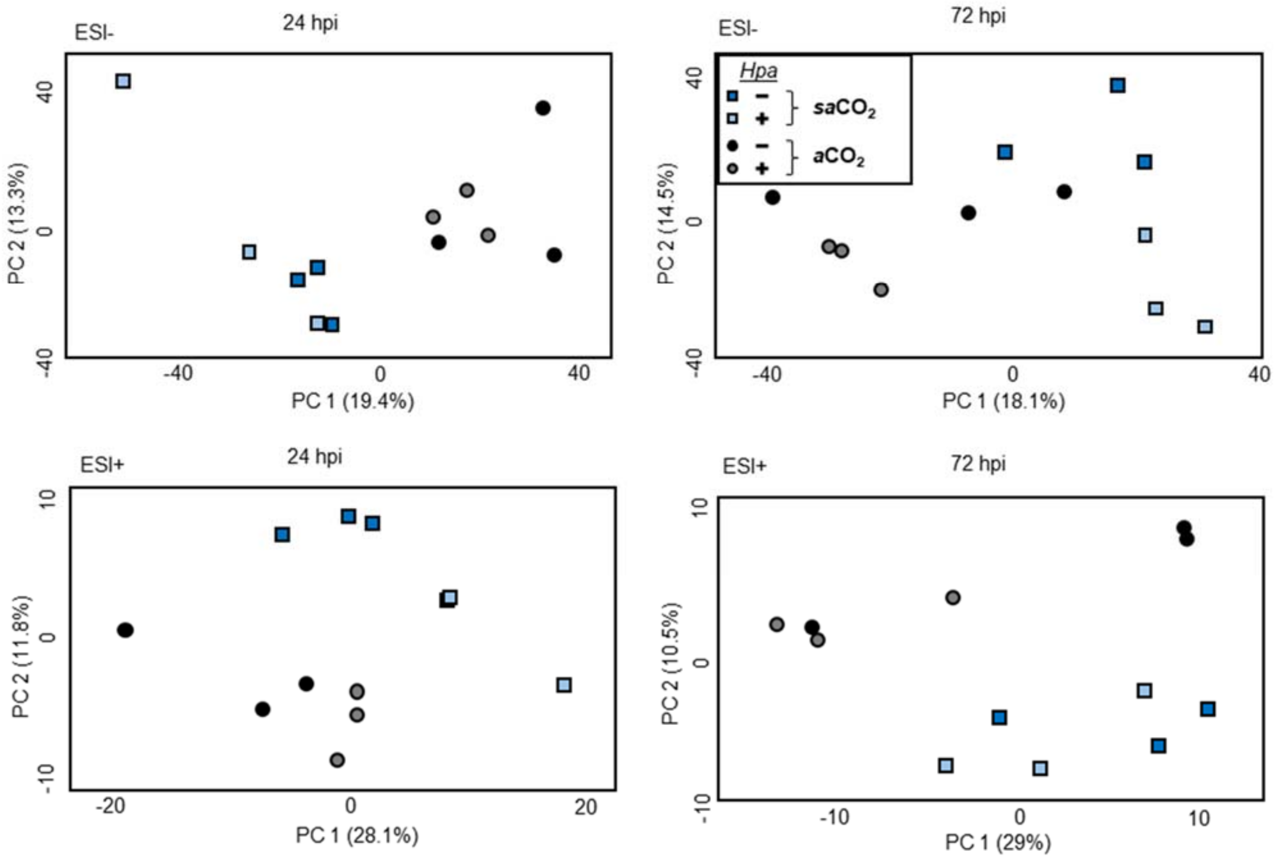

Fig. S5 Global metabolic signatures of mock- and *Hpa*-inoculated Arabidopsis (Col-0) at the 8-leaf growth stage at *sa*CO_2 (200 ppm) and *a*CO_2 (400 ppm). Shown are principal component analysis (PCA) plots of negative (ESI^-; 4497 ions) and positive (ESI^+; 5683 ions) ionizations, obtained by UPLC-Q-TOF analysis of methanol extracts from leaf tissue at 24 and 72 hpi. Samples from plants grown at *sa*CO_2 are indicated by circles; samples from plants at *a*CO_2 are indicated by squares. Black/blue symbols indicate mock-inoculated plants; grey/ light blue symbols indicate samples from *Hpa*-inoculated plants.

(a)

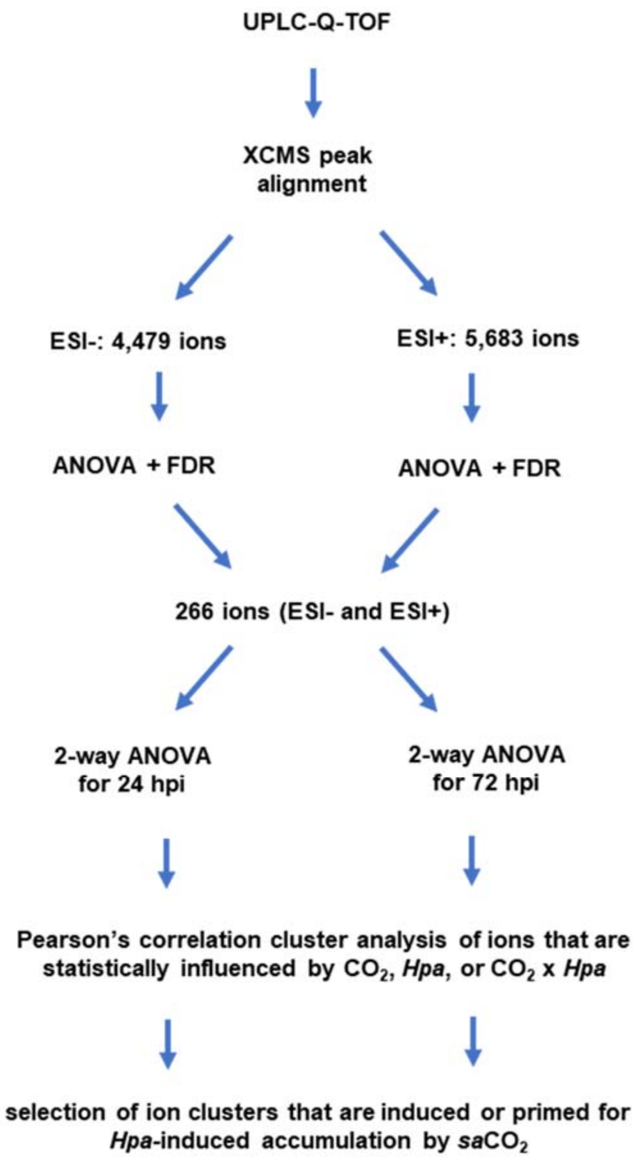

(b)

Factor	24 hpi	72 hpi
CO_2	111	57
Hpa	65	34
CO_2 x *Hpa*	24	17

(c)

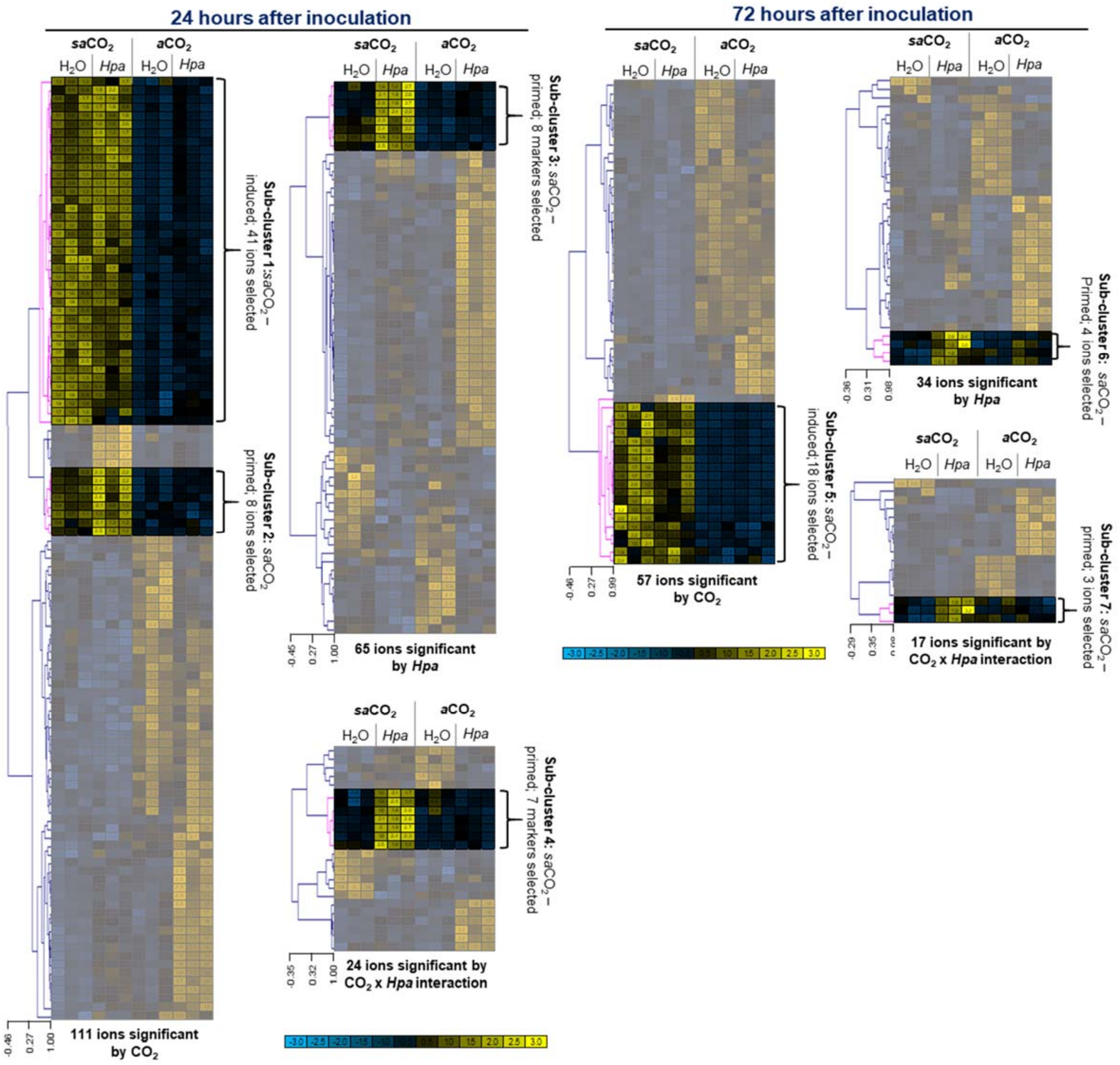

Fig S6 Selection of ions that are induced or primed for *Hpa*-induced accumulation by *sa*CO_2. (a) Schematic pipeline of the selection procedure. (b) Numbers of statistically significant ions by 2-way ANOVA of the selection of 266 ions. (c) Hierarchical cluster analysis (Pearson's correlation) of ions that are significantly influenced by CO_2, *Hpa* or the interaction CO_2 x *Hpa*. Highlighted are ion clusters showing direct induction (*sa*CO_2 - induced) or priming for *Hpa*-induced accumulation by *sa*CO_2 (*sa*CO_2 - primed).

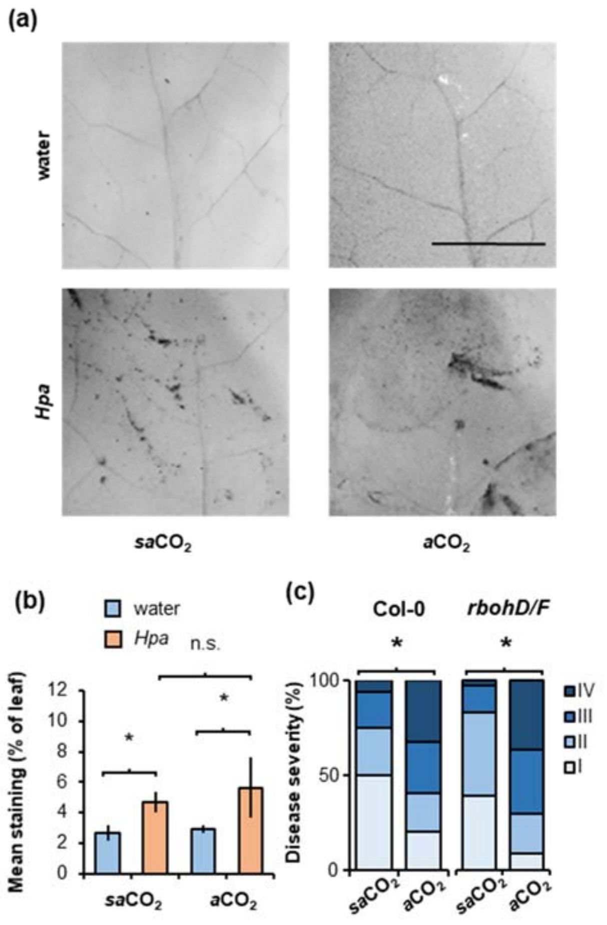

Fig. S7 Extracellular H_2O_2 in *sa*CO_2-induced resistance against *Hpa*. (a) Visualization of extracellular H_2O_2 accumulation in leaves of Arabidopsis (Col-0) at *a*CO_2 (400 ppm) and *sa*CO2 (200 ppm). Shown are 3,3′-diaminobenzidine (DAB)-stained leaves at 48 hours after mock (water) or *Hpa* inoculation. Bar = 1 mm. (b) Quantification of DAB staining signal by image analysis. Shown are mean values of the stained proportion of the leaf area (± SD, n = 5). (c) Evaluation of *Hpa* resistance at *a*CO_2 and *sa*CO_2 in Col-0 and *rbohD/F* plants at the 8-leaf stage. The *rbohD/F* double mutant is impaired in production of extracellular H_2O_2 by NADP-dependent oxidase. Shown are relative numbers of leaves (n > 50) in *Hpa* colonization classes of increasing severity (I–IV) at 7 dpi. Asterisks indicate statistically significant differences between CO_2 conditions (Fisher's exact test; $P < 0.05$).

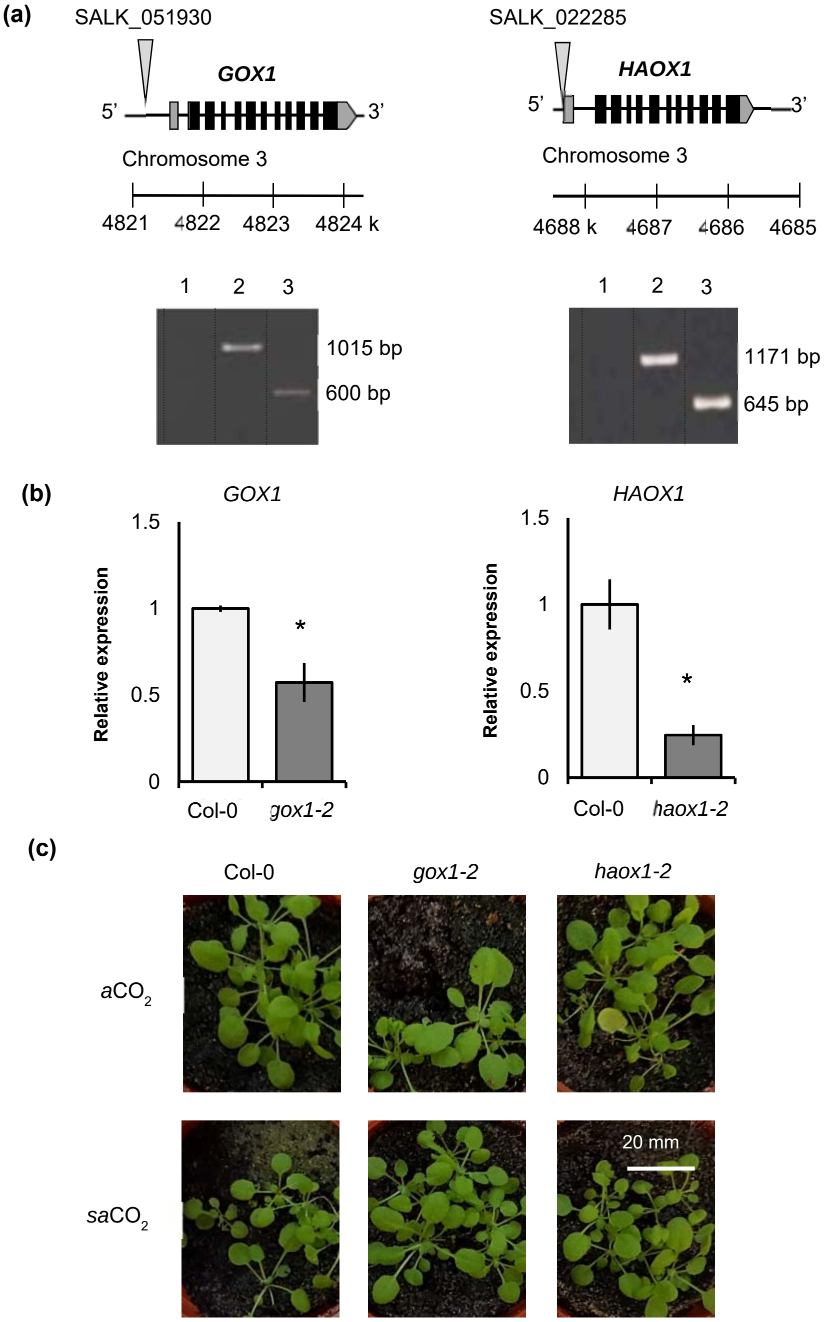

Fig. S8. Selection of *gox1-2* (SALK_051930) and *haox1-2* (SALK_022285) *knock-down* mutants. (a) PCR confirmation of homozygous T-DNA insertions. Gene models show locations of T-DNA insertions in promoter regions of *GOX1* and *HAOX1*. Images show PCR products from 1) mutant DNA with LP + RP primers (no band); 2) Col-0 DNA with LP + RP primers, and 3), mutant DNA with LBb1.3 + RP primers. (b) Impacts of *knock-down* mutations on transcription of *GOX1* and *HAOX1* in *gox1-2* and *haox1-2* plants, respectively. Shown are mean values of relative transcript levels (± SD; n = 5) in shoot tissues of 3-week old plants. Asterisks indicate statistically significant reductions in relative transcript level compared to wild-type plants (Col-0; Student's t-test, $P < 0.05$). The experiment was repeated with similar results. (c) Growth phenotypes of 3-week old Col-0, *gox1-2* and *haox1-2* at aCO_2 and $saCO_2$ conditions.

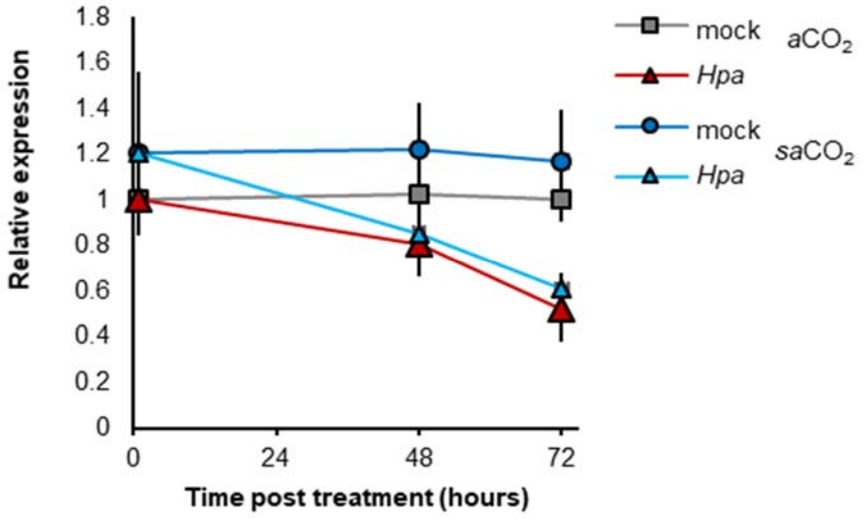

Fig. S9. Impacts of *Hpa* inoculation on *CAT2* gene expression in 8-leaf Col-0 plants at *sa*CO_2 (200 ppm) and *a*CO_2 (400 ppm). Shown are mean values of relative transcript abundance (± SD, n = 5) at different hours post mock (water) or *Hpa* inoculation.

Table S1. Primers used for mutant genotyping and RT-qPCR analysis of gene expression

	Gene	Gene locus	Forward primer sequence (5' - 3')	Reverse primer sequence (5' - 3')
**Genotyping**	*GOX1*	AT3G14420	CCG AAA GCT ATT AAA CAG CCC	CTT ACA TTG CAC CCA ACT TCC
	HAOX1	AT3G14130	GCA GAA TGG AGG GGT TTA GTC	CAT GCA AGA ATC TTG CTC CTC
	SALK Insert (LBb1.3)		-	ATT TTG CCG ATT TCG GAA C
**qPCR primers**	Gene expression			
	GOX1	AT3G14420	AGA ACA GCA GCA ACA CAG AAC	CAC TAG GCT TGG TTT GTG ATC TGA TA
	HAOX1	AT3G14130	GAA TTA AAT CTA TGC TCT GAT CCT AAA ACC	GAA CAA GTC CAA CGT ACT ATT GTC TT
	CAT2	AT4G35090	CGA GGT ATG ACC AGG TTC GT	CTT CCA GGC TCC TTG AAG TTG
	PR1	AT2G14610	GTC TCC GCC GTG AAC ATG T	CGT GTT CGC AGC GTA GTT GT
	VSP2	AT5G24770	GGA CTT GCC CTA AAG AAC GAC ACC	GTC GGT CTT CTC TGT TCC GTA TCC
	UBC 9	AT5G25760	TCA CAA TTT CCA AG^ GTG CTG C	TCA TCT GGG TTT GGA TCC GT
	SAND	AT2G28390	AAC TCT ATG CAG CAT T	GGT GGT ACT AGC ACA A
	DNA quantification			
	HpaACT	ID807716	GTG TCG CAC ACT GTA CCC ATT TAT	ATC TTC ATC ATG TAG TCG GTC AAG T
	Pcβ-Tub		CAA GTA TGT TCC CCG AGC CGT	GAA GAG CTG ACC GAA GGG ACC
	AtACT2	AT3G18780	AAT CAC AGC ACT TGC ACC A	GAG GGA AGC AAG AAT GGA AC

Table S2. Putative identification of metabolic markers detected by UPLC-Q-TOF

sample [a]	*P* value [b]	Detected *m/z* [c]	RT (min) [c]	*Adducts* [d]	Predicted mass [d]	Error (ppm) [d]	Putative compound [d]	Predicted formula [d]	Pathways [e]
**24 hours**	1.2E-03	351.992	4.4	*[M+K]+*	313.021	19	Eudistomin H	C15H12BrN3	Alkaloids
	6.0E-03	422.166	1.6	*[M-H]-*	423.168	13	N-Methyl-2,3,7,8-tetramethoxy-5,6-dihydrobenzophenanthridine-6-ethanoic acid	C24H25NO6	Alkaloids
	6.6E-03	482.067	1.8	*[M+Cl]-*	447.108	22	Pigment A aglycone	C25H19O8	Anthocyanins
	1.0E-03	244.027	1.5	*[M+K-2H]-*	207.081	11	Anthocyanidins	C15H11O	Anthocyanins
	2.2E-04	933.074	1.1	*[M-H]-*	934.071	10	Vescalagin	C41H26O26	Anthocyanins
	2.2E-04	391.066	1.1	*[M+Na-2H]-*	370.090	4	5-Hydroxy-6-methoxycoumarin 7-glucoside	C16H18O10	Coumarin
	2.2E-04	457.133	2.9	*[M-H]-*	458.142	5	cis-p-Coumaric acid 4-[apiosyl-(1->2)-glucoside]	C20H26O12	Coumarin
	5.3E-03	427.124	1.5	*[M+Na]+*	404.126	20	Calomelanol C	C24H20O6	Flavonoids
	1.3E-03	329.008	4.4	*[M+2Na-H]+*	284.032	13	7,4\',5\'-Trihydroxy-5,2\'-oxido-4-phenylcoumarin	C15H8O6	Flavonoids
	6.0E-03	639.161	3.0	*[M-H]-*	640.164	6	Laricitrin 3-rutinoside	C28H32O17	Flavonoids
	9.0E-03	932.246	2.8	*[M-H]-*	933.266	14	Pelargonidin 3-O-[b-D-Glucopyranosyl-(1->2)-[4-hydroxy-3-methoxy-(E)-cinnamoyl-(->6)]-b-D-glucopyranoside] 5-O-b-D-glucopyranoside	C43H49O23	Flavonoids
	2.5E-03	421.163	1.6	*[M-H]-*	422.173	6	Euchrenone b10	C25H26O6	Flavonoids
	5.6E-03	487.123	2.7	*[M-H]-*	488.132	3	Acacetin 7-(2\'\'-acetylglucoside)	C24H24O11	Flavonoids
	1.4E-03	667.080	3.4	*[M+2Na-H]+*	622.117	12	Apigenin 7-glucuronosyl-(1->2)-glucuronide	C27H26O17	Flavonoids
	3.4E-03	603.292	1.6	*[M-H]-*	604.288	10	Cerbertin	C32H44O11	Glucosides
	7.8E-04	199.097	2.5	*[M-H]-*	200.105	2	Decenedioic acid	C10H16O4	Lipids
	8.2E-03	453.209	3.0	*[M+Na-2H]-*	432.236	2	Glucosyl (2E,6E,10x)-10,11-dihydroxy-2,6-farnesadienoate	C21H36O9	Lipids
	8.4E-03	869.480	5.5	*[M+Cl]-*	833.521	20	PS (18:1(9Z)/22:6(4Z,7Z,10Z,13Z,16Z,19Z))	C46H76NO10P	Lipids
	6.0E-03	603.289	1.6	*[M+Cl]-*	567.317	8	PS (10:0/10:0)	C26H50NO10P	Lipids
	4.2E-03	449.190	1.6	*[M+K-2H]-*	411.239	10	PC (O-8:0/2:0)	C18H38NO7P	Lipids
	8.3E-03	431.196	1.6	*[M+Cl]-*	397.223	7	PE (12:0/0:0)	C17H36NO7P	Lipids
	1.4E-03	251.003	4.4	*[M+H]+*	250.000	18	Glycerate	C6H10CaO8	Photorespiration
	3.9E-03	315.108	2.5	*[M-H]-*	316.116	3	Hydroxytyrosol 1-O-glucoside	C14H20O8	Polyphenol
	2.7E-04	249.112	3.4	*[M+H]+*	248.105	0	Prenyl caffeate	C14H16O4	Polyphenol
	1.2E-04	350.988	4.4	*[M+K]+*	312.022	7	Gamma-Glutamyl-Se-methylselenocysteine	C9H16N2O5Se	Redox
	4.4E-03	267.087	1.6	*[M-H]-*	268.088	21	Cysteinyl-Phenylalanine	C12H16N2O3S	Redox
	6.9E-04	665.080	3.3	*[M+H]+*	664.093	30	Deamino-NAD^{+}	C21H26N6O15P2	Redox
	1.1E-03	493.077	2.9	*[M+Na]+*	470.088	0	Phe4Cl-Tyr-OH	C23H19ClN2O7	Amino acids
	6.6E-03	423.159	1.6	*[M+Na-2H]-*	402.189	10	D-Linalool 3-(6''-malonylglucoside)	C19H30O9	Terpenoids
	5.1E-04	467.166	2.1	*[M-H]-*	468.178	11	Dukunolide D	C26H28O8	Terpenoids
	4.2E-03	431.192	1.6	*[M-H]-*	432.197	5	S-Furanopetasitin	C24H32O5S	Terpenoids
	1.1E-03	331.001	4.4						Unknown
	7.3E-03	330.987	4.4						Unknown
	1.1E-04	97.978	4.4						Unknown
	8.1E-03	330.957	4.4						Unknown
	3.0E-05	249.986	4.4						Unknown
	1.7E-03	997.443	2.9						Unknown
	7.4E-03	1001.458	2.8						Unknown
	8.2E-03	731.210	1.9						Unknown
	1.4E-03	1100.454	3.0						Unknown
	8.1E-03	1187.442	2.8						Unknown
**72 hours**	5.5E-03	634.415	6.2	*[M+NH4]+*	616.378	5	Tabernamine	C40H48N4O2	Alkaloids
	1.3E-03	329.008	4.4	*[M+2Na-H]+*	284.032	13	7,4\',5\'-Trihydroxy-5,2\'-oxido-4-phenylcoumarin	C15H8O6	Coumarin
	1.1E-03	493.077	2.9	*[M+2Na-H]+*	448.101	10	1,2,6,8-Tetrahydroxy-3-methylanthraquinone 2-O-b-D-glucoside	C21H20O11	Flavonoids
	2.2E-04	391.066	1.1	*[M-H]-*	392.074	2	5,7,3\',4\',5\'-Pentahydroxy-3,6,8-trimethoxyflavone	C18H16O10	Flavonoids
	5.3E-03	427.124	1.5	*[M+H-H2O]+*	444.121	13	Artomunoxanthentrione	C26H20O7	Flavonoids
	2.5E-03	421.163	1.6	*[M-H]-*	422.173	6	Euchrenone b10	C25H26O6	Flavonoids
	8.1E-03	348.991	4.4	*[M+2Na-H]+*	304.014	16	6-Chloroapigenin	C15H9ClO5	Flavonoids
	8.2E-03	730.203	1.9	*[M+NH4]+*	712.185	21	Syringetin 3-(,6\'\'\'-acetylglucosyl)(1->6)-galactoside	C31H36O19	Flavonoids
	1.0E-03	244.027	1.5	*[M+H+Na]2+*	464.056	1	4-Hydroxyglucobrassicin	C16H20N2O10S2	Glucosinolate
	1.4E-03	664.066	3.4	*[M+Na]+*	641.091	20	6-Sinapoylglucoraphenin	C23H31NO14S3	Glucosinolate
	1.4E-03	251.003	4.4	*[M+H]+*	250.000	18	Glycerate	C6H10CaO8	Photorespiration
	7.4E-03	301.119	1.7	*[M+Na-2H]-*	280.142	5	Feruloyl-2-hydroxyputrescine	C14H20N2O4	Polyamines
	1.2E-04	350.988	4.4	*[M+K]+*	312.022	7	Gamma-Glutamyl-Se-methylselenocysteine	C9H16N2O5Se	Redox
	6.9E-04	664.071	3.3	*[M+H]+*	663.109	68	NAD^{+}	C21H27N7O14P2	Redox
	1.1E-03	331.001	4.4						Unknown
	7.3E-03	330.987	4.4						Unknown
	1.2E-03	327.999	4.4						Unknown
	1.1E-04	97.978	4.4						Unknown
	3.0E-05	249.986	4.4						Unknown
	2.0E-04	992.945	8.7						Unknown
**24 hours + *Hpa***	9.95E-03	165.080	0.8	*[M+H-2H2O]+*	200.095	16	Harmalol	C12H12N2O	Alkaloids
	9.9E-03	882.307	3.0	*[M-H]-*	883.290	27	Wilfordine	C43H49NO19	Alkaloids
	4.0E-05	293.100	1.3	*[M-H]-*	294.106	3	N-Glycosyl-L-asparagine	C10H18N2O8	Amino acids
	1.6E-03	416.105	2.3	*[M-H]-*	417.117	11	Asn-TyrMe-OH	C19H19N3O8	Amino acids
	5.3E-03	146.061	1.0	*[M+H-2H2O]+*	181.074	1	L-Tyrosine	C9H11NO3	Amino acids
	3.6E-03	778.169	2.3	*[M+Na-2H]-*	757.219	32	Pelargonidin 3-sophoroside 5-glucoside	C33H41O20	Flavonoids

	1.0E-05	189.074	1.8	*[M-H2O-H]-*	208.089	17	Chalcone	C15H12O	Flavonoids
	1.1E-03	417.107	2.3	*[M-H]-*	418.126	29	4\'-Hydroxy-3,5,6,7,3\',5\'-hexamethoxyflavone	C21H22O9	Flavonoids
	8.0E-03	557.298	2.9	*[M-H]-*	558.298	12	Denticulaflavonol	C35H42O6	Flavonoids
	1.7E-03	454.203	3.0	*[M-H]-*	455.212	2	5-Ribosylparomamine	C17H33N3O11	Glucosides
	6.4E-04	402.089	1.7	*[M-H]-*	403.097	1	3-Methylpentyl glucosinolate	C13H25NO9S2	Glucosinolates
	5.4E-03	448.069	1.7	*[M+2Na-H]+*	403.097	0	3-Methylpentyl glucosinolate	C13H25NO9S2	Glucosinolates
	2.7E-04	249.112	3.4	*[M+Na-2H]-*	228.136	5	(-)-11-hydroxy-9,10-dihydrojasmonic acid	C12H20O4	Phytohormones
	1.12E-03	119.074	0.8	*[M+H-H2O]+*	136.0749	15	Tetrahydropteridine	C6H8N4	Redox
	3.6E-03	221.983	0.4	*[M-H2O-H]-*	241.008	29	3-Mercaptolactate-cysteine disulfide	C6H11NO5S2	Thiols
	2.6E-04	1044.112	5.4						Unknown
**72 hours + *Hpa***	7.4E-03	301.119	1.7	*[M-H]-*	302.115	36	2,4\'-Dihydroxy-4,6-dimethoxydihydrochalcone	C17H18O5	Flavonoids
	8.3E-03	543.245	4.3	*[M+Na-2H]-*	522.268	4	7,8-Dihydro-3b,6a-dihydroxy-alpha-ionol 9-[apiosyl-(1->6)-glucoside]	C24H42O12	Flavonoids
	5.4E-03	448.069	1.7	*[M+2Na-H]+*	403.097	1	3-Methylpentyl glucosinolate	C13H25NO9S2	Glucosides
	5.7E-03	525.353	5.8	*[M+H]+*	524.348	3	PG (P-20:0/0:0)	C26H53O8P	Lipids
	1.5E-03	547.590	6.2						Unknown
	1.8E-03	518.763	5.6						Unknown

[a] conditions for which the metabolic markers showed a statistically significant accumulation between saCO2 and aCO2
[b] P values indicate levels of significance from FDR adjusted ANOVA and subsequent two-factor ANOVA ($P < 0.01$)
[c] accurate m/z values with their corresponding retention time (RT) detected by UPLC-qTOF-MS
[d] predicted parameters from the METLIN database using the detected accurate m/z
[e] putative metabolites and their corresponding pathways were validated by information from the PubMed chemical database
* putative metabolites that are oxidised
Adducts : type of ion generated by electrospray ionization; Δppm: difference between observed and theoretical monoisotopic masses; PC: Phosphatidylcholine; PE: Phosphatidylethanolamine; PG: Phosphoglycerol; PS: Phosphatidylserine.

Methods S1: supplemental Materials and Methods

Chemicals and reagents. All chemicals and solvents (higher analytical MS grade) used in this study were purchased from Sigma-Aldrich (UK), except JA which was obtained from OlChemim (http://www.olchemim.cz/).

Targeted quantification of hormones. SA and JA were quantified by UPLC-Q-TOF-MS^E, using a previous method (Pétriacq *et al.*, 2016). Briefly, phytohormones were double- extracted from frozen leaf material (10 mg dry weight) in a total volume of 1.5 mL of ethyl acetate. Each biologically replicated sample consisted of 4 pooled leaves of similar size and age from different plants. Hormones were quantified by UPLC-Q-TOF-MS^E in negative electrospray ionization mode (ESI$^-$), using standard curves of pure SA and JA. Compound identity was verified by the following fragmentation patterns: SA, 137→93; and JA, 209→59.

Untargeted metabolic profiling by UPLC-qTOF-MS^E. Plant metabolic profiles at *sa*CO_2 or *a*CO_2 were analysed after application of DC. Plants in the 8-leaf stage were inoculated with *Hpa* or water (mock), after which leaf tissue of 4 plants from each pot were pooled as one biological replicate. Replicates (n = 3) were collected in the middle of the photoperiod and snap frozen in liquid nitrogen at 24 hpi and 72 hpi. UPLC-Q-TOF-MS^E analysis of methanol extracts was carried out as described previously (Pétriacq *et al.*, 2016), using the following modifications: high-resolution full-scan mass spectrometry was performed with a SYNAPT G2 HDMS Q-TOF mass spectrometer (Waters), coupled to a UPLC BEH C18 column (2.1 × 50 mm, 1.7 µm, Waters) with a guard column (VanGuard, 2.1 x 5 mm, 1.7 µm, Waters) for separation of compounds at a flow rate of 400 µL min^{-1}. The mobile phase consisted of A; water with 0.05% formic acid, and B; acetonitrile with 0.05% formic acid with a gradient applied: 0 – 3 min 5 – 35 % B, 3 – 6 min 35 – 100 % B, holding at 100 % B for 2 min, 8 – 10 min, 100 – 5 % B. The column temperature was kept at 45 °C with an injection volume of 10 µL. Buffer (50% methanol) was injected between treatments and between ESI$^-$ and ESI$^+$ ionization modes for stabilization of the electrospray ionization source.

Ions were detected over a mass range of 50 – 1200 Da, using a scan time of 0.2 s (ESI$^-$ and ESI$^+$) with the instrument operating in sensitivity mode for the MS full scan (*i.e.* without collision energy). Collision energy was ramped in the transfer cell from 5 to 45 eV (MS^E), using the following conditions:

	**ESI$^-$**	**ESI$^+$**
Capillary voltage (kV)	- 3	+ 3
Sampling cone voltage (V)	- 25	+ 25
Extraction cone voltage (V)	- 4.5	+ 10

Source Temperature (°C)	120	120
Desolvation Temperature (°C)	350	350
Desolvation gas flow (L h^{-1})	800	800
Cone gas flow (L h^{-1})	60	60

Prior to analysis, the Q-TOF detector was calibrated with a solution of sodium formate. During each run, accurate mass measurements were ensured by infusing leucine enkephalin peptide as an internal reference (10 s scan frequency, cone voltage of 40 V and a capillary voltage of 3 kV). The system was controlled by MassLynx v 4.1 software (Waters).

Statistical analysis of metabolic profiling data. Raw files obtained from MassLynx were converted into CDF format, using the Databridge function in MassLynx (v. 4.1). Subsequent alignment and integration of metabolic peaks were performed in R (v 3.1.3), using XCMS (Smith *et al.*, 2006). Peaks were retained for analysis when present in all bioreplicates (k = 3), at a threshold intensity of 10 (I = 10) and at maximum resolution range of 20 ppm. Peak values from each run were normalised for total ion current (TIC). For each sample, normalised peak values were corrected for dry weight, generating a dataset of 5683 *m/z* values in ESI^+ and 4479 *m/z* values in ESI^-.

Global differences in metabolic signals between treatment/point combinations was visualised for anions (ESI^-) and cations (ESI^+) separately by principal component analysis (PCA, Fig. S5), using MetaboAnalyst online (v. 3.0; http://www.metaboanalyst.ca; Xia et al., 2015) on median-normalised, cube-root-transformed and Pareto-scaled data. To select ions that are induced by *sa*CO_2 directly or primed by *sa*CO_2 for augmented induction following *Hpa* inoculation, ESI^- and ESI^+ datasets were analysed separately for statistically significant differences between all CO_2/treatment/time-point combinations by one-way ANOVA ($P < 0.01$ + Benjamini-Hochberg false discovery rate correction, FDR; see Fig. S6), using MarVis (v. 1.0; http://marvis.gobics.de; Kaever et al., 2012). From each ionization mode, 133 statistically significant markers were combined into one dataset of 266 markers for successive 2-way ANOVA ($P < 0.01$), using MeV (v. 4.9.0; http://mev.tm4.org). Heatmaps project TIC- normalised ion current (NIC) values, relative to the average and standard deviation of all NIC values across all samples: Value = (NIC – mean) / SD. For each time-point (24 and 72 hpi), this analysis resulted in 3 subsets of markers, whose intensity was influenced by CO_2, *Hpa*, or the CO_2 x *Hpa* interaction (Fig. S6b). Hierarchal clustering (Pearson′s correlation; MeV) allowed visual selection of ion clusters that are induced directly by *sa*CO_2 or primed for augmented induction after *Hpa* inoculation, as detailed in Fig. S6c. Putative identities of the selected ion markers were based on *m/z* values at stringent accuracy (< 30 ppm), the using METLIN chemical database (Smith *et al.*, 2005).

References

Kaever A, Landesfeind M, Possienke M, Feussner K, Feussner I, Meinicke P. **2012**. MarVis-Filter: ranking, filtering, adduct and isotope correction of mass spectrometry data. *Journal of biomedicine & biotechnology* **2012**: 263910.

Pétriacq P, Ton J, Patrit O, Tcherkez G, Gakière B. **2016**. NAD acts as an integral regulator of multiple defense layers. *Plant Physiology* **172**: 1465–1479.

Smith CA, O'Maille G, Want EJ, Qin C, Trauger SA, Brandon TR, Custodio DE, Abagyan R, Siuzdak G. **2005**. METLIN: a metabolite mass spectral database. *Therapeutic Drug Monitoring* **27**: 747–751.

Smith CA, Want EJ, O'Maille G, Abagyan R, Siuzdak G. **2006**. XCMS: processing mass spectrometry data for metabolite profiling using nonlinear peak alignment, matching, and identification. *Analytical Chemistry* **78**: 779–787.

Xia J, Sinelnikov I V., Han B, Wishart DS. **2015**. MetaboAnalyst 3.0—making metabolomics more meaningful. *Nucleic Acids Research* **43**: W251–W257.
